# Neuroanatomical heterogeneity and homogeneity in individuals at clinical high risk for psychosis

**DOI:** 10.1038/s41398-022-02057-y

**Published:** 2022-07-26

**Authors:** Helen Baldwin, Joaquim Radua, Mathilde Antoniades, Shalaila S. Haas, Sophia Frangou, Ingrid Agartz, Paul Allen, Ole A. Andreassen, Kimberley Atkinson, Peter Bachman, Inmaculada Baeza, Cali F. Bartholomeusz, Michael W. L. Chee, Tiziano Colibazzi, Rebecca E. Cooper, Cheryl M. Corcoran, Vanessa L. Cropley, Bjørn H. Ebdrup, Adriana Fortea, Louise Birkedal Glenthøj, Holly K. Hamilton, Kristen M. Haut, Rebecca A. Hayes, Ying He, Karsten Heekeren, Michael Kaess, Kiyoto Kasai, Naoyuki Katagiri, Minah Kim, Jochen Kindler, Mallory J. Klaunig, Shinsuke Koike, Alex Koppel, Tina D. Kristensen, Yoo Bin Kwak, Jun Soo Kwon, Stephen M. Lawrie, Irina Lebedeva, Jimmy Lee, Ashleigh Lin, Rachel L. Loewy, Daniel H. Mathalon, Chantal Michel, Romina Mizrahi, Paul Møller, Barnaby Nelson, Takahiro Nemoto, Dorte Nordholm, Maria A. Omelchenko, Christos Pantelis, Jayachandra M. Raghava, Jan I. Røssberg, Wulf Rössler, Dean F. Salisbury, Daiki Sasabayashi, Ulrich Schall, Lukasz Smigielski, Gisela Sugranyes, Michio Suzuki, Tsutomu Takahashi, Christian K. Tamnes, Jinsong Tang, Anastasia Theodoridou, Sophia I. Thomopoulos, Alexander S. Tomyshev, Peter J. Uhlhaas, Tor G. Værnes, Therese A. M. J. van Amelsvoort, Theo G. M. Van Erp, James A. Waltz, Lars T. Westlye, Stephen J. Wood, Juan H. Zhou, Philip McGuire, Paul M. Thompson, Maria Jalbrzikowski, Dennis Hernaus, Paolo Fusar-Poli, the ENIGMA Clinical High Risk for Psychosis Working Group, Camilo de la Fuente-Sandoval, Sabrina Catalano, Daniela Hubl, Jason Schiffman, Enea D. Venegoni, Christine I. Hooker, Paul E. Rasser, Wenche ten Velden Hegelstad, Franz Resch, Imke L. J. Lemmers-Jansen, G. Paul Amminger, Xiaogang Chen, Kang Ik K. Cho, Birte Yding Glenthøj, Lieuwe de Haan, Matthew A. Harris, Wu Jeong Hwang, Pablo León-Ortiz, Xiaoqian Ma, Patrick McGorry, Ricardo Mora-Durán, Masafumi Mizuno, Merete Nordentoft, Lijun Ouyang, Jose C. Pariente, Francisco Reyes-Madrigal, Mikkel E. Sørensen, Dennis Velakoulis, Sophia Vinogradov, Christina Wenneberg, Hidenori Yamasue, Liu Yuan, Alison R. Yung

**Affiliations:** 1 Early Psychosis: Interventions and Clinical-detection (EPIC) Lab, Department of Psychosis Studies, Institute of Psychiatry, Psychology & Neuroscience, King’s College London, London, UK; 2 National Institute for Health Research, Maudsley Biomedical Research Centre, South London and Maudsley NHS Foundation Trust, London, UK; 3 Institut d’Investigacions Biomèdiques August Pi i Sunyer, CIBERSAM, Barcelona, Spain; 4 Department of Clinical Neuroscience, Karolinska Institutet, Stockholm, Sweden; 5 Department of Psychiatry, Icahn School of Medicine at Mount Sinai, New York City, NY USA; 6 Department of Psychiatry, University of British Columbia, Vancouver, BC Canada; 7 Department of Psychiatric Research, Diakonhjemmet Hospital, Oslo, Norway; 8 Centre for Psychiatry Research, Department of Clinical Neuroscience, Karolinska Institutet & Stockholm Health Care Services, Stockholm County Council, Stockholm, Sweden; 9 Norwegian Centre for Mental Disorders Research, Institute of Clinical Medicine, University of Oslo, Oslo, Norway; 10 KG Jebsen Center for Neurodevelopmental Disorders, University of Oslo, Oslo, Norway; 11 Department of Psychology, University of Roehampton, London, UK; 12 Department of Psychosis Studies, Institute of Psychiatry, Psychology and Neuroscience, King’s College London, London, UK; 13 NORMENT, Division of Mental Health and Addiction, Oslo University Hospital & Institute of Clinical Medicine, University of Oslo, Oslo, Norway; 14 Division of Psychiatry, University of Edinburgh, Edinburgh, UK; 15 Department of Psychiatry, University of Pittsburgh, Pittsburgh, PA USA; 16 Department of Child and Adolescent Psychiatry and Psychology, Institute of Neuroscience, 2017SGR-881, Hospital Clinic Barcelona, Institut d’Investigacions Biomèdiques August Pi i Sunyer (IDIBAPS), Centro de Investigación Biomédica en Red de Salud Mental (CIBERSAM), Universitat de Barcelona, Barcelona, Spain; 17 Centre for Youth Mental Health, University of Melbourne, Melbourne, VIC Australia; 18 Orygen, Melbourne, VIC Australia; 19 Center for Sleep and Cognition, Yong Loo Lin School of Medicine, National University of Singapore, Singapore, Singapore; 20 Department of Psychiatry, Columbia University, New York City, NY USA; 21 New York State Psychiatric Institute, New York City, NY USA; 22 Melbourne Neuropsychiatry Centre, Department of Psychiatry, University of Melbourne & Melbourne Health, Carlton South, VIC Australia; 23 Mental Illness Research, Education, and Clinical Center, James J Peters VA Medical Center, New York City, NY USA; 24 Centre for Mental Health, Faculty of Health, Arts and Design, School of Health Sciences, Swinburne University, Melbourne, VIC Australia; 25 Centre for Neuropsychiatric Schizophrenia Research (CNSR), Mental Health Centre Glostrup, Copenhagen University Hospital, Glostrup, Denmark; 26 Department of Clinical Medicine, Faculty of Health and Medical Sciences, University of Copenhagen, Copenhagen, Denmark; 27 Department of Child and Adolescent Psychiatry and Psychology, Institute of Neuroscience, Hospital Clinic Barcelona, Fundació Clínic Recerca Biomèdica, Universitat de Barcelona, Barcelona, Spain; 28 Copenhagen Research Center for Mental Health, Mental Health Center Copenhagen, University of Copenhagen, Copenhagen, Denmark; 29 Department of Psychiatry and Behavioral Sciences, University of California San Francisco, San Francisco, CA USA; 30 San Francisco Veterans Affairs Health Care System, San Francisco, CA USA; 31 Department of Psychiatry and Behavioral Sciences, Rush University Medical Center, Chicago, IL USA; 32 National Clinical Research Center for Mental Disorders and Department of Psychiatry, The Second Xiangya Hospital of Central South University, Changsha, Hunan China; 33 Department of Psychiatry and Psychotherapy I, LVR-Hospital Cologne, Cologne, Germany; 34 Department of Psychiatry, Psychotherapy and Psychosomatics, Psychiatric University Hospital Zurich, University of Zurich, Zurich, Switzerland; 35 Department of Child and Adolescent Psychiatry, Center of Psychosocial Medicine, University of Heidelberg, Heidelberg, Germany; 36 University Hospital of Child and Adolescent Psychiatry and Psychotherapy, University of Bern, Bern, Switzerland; 37 Department of Neuropsychiatry, Graduate School of Medicine, The University of Tokyo, Tokyo, Japan; 38 The University of Tokyo Institute for Diversity and Adaptation of Human Mind, Tokyo, Japan; 39 The International Research Center for Neurointelligence at The University of Tokyo Institutes for Advanced Study, The University of Tokyo, Tokyo, Japan; 40 Department of Neuropsychiatry, Toho University School of Medicine, Tokyo, Japan; 41 Department of Neuropsychiatry, Seoul National University Hospital, Seoul, Republic of Korea; 42 Department of Psychiatry, Seoul National University College of Medicine, Seoul, Republic of Korea; 43 Department of Psychology, University of Maryland, Baltimore County, MD USA; 44 Center for Evolutionary Cognitive Sciences, Graduate School of Art and Sciences, The University of Tokyo, Tokyo, Japan; 45 Department of Pharmacology and Toxicology, University of Toronto, Toronto, ON Canada; 46 Department of Brain and Cognitive Sciences, Seoul National University College of Natural Sciences, Seoul, Republic of Korea; 47 Department of Psychosis, Institute of Mental Health, Singapore, Singapore; 48 Laboratory of Neuroimaging and Multimodal Analysis, Mental Health Research Center, Moscow, Russian Federation; 49 Lee Kong Chian School of Medicine, Nanyang Technological University, Singapore, Singapore; 50 Telethon Kids Institute, The University of Western Australia, Perth, WA Australia; 51 Douglas Research Center, Montreal, QC Canada; 52 Department of Psychiatry, McGill University, Montreal, QC Canada; 53 Department for Mental Health Research and Development, Division of Mental Health and Addiction, Vestre Viken Hospital Trust, Drammen, Norway; 54 Department of Youth Psychiatry, Mental Health Research Center, Moscow, Russian Federation; 55 Florey Institute of Neuroscience and Mental Health, Center for Mental Health, Parkville, VIC Australia; 56 Department of Clinical Physiology, Nuclear Medicine and PET, Functional Imaging Unit, University of Copenhagen, Glostrup, Denmark; 57 Centre for Neuropsychiatric Schizophrenia Research, Mental Health Centre Glostrup, University of Copenhagen, Glostrup, Denmark; 58 Department of Psychiatry and Psychotherapy, Charité Universitätsmedizin Berlin, Berlin, Germany; 59 Department of Neuropsychiatry, University of Toyama Graduate School of Medicine and Pharmaceutical Sciences, Toyama, Japan; 60 Research Center for Idling Brain Science, University of Toyama, Toyama, Japan; 61 Priority Centre for Brain and Mental Health Research, The University of Newcastle, Newcastle, NSW Australia; 62 Priority Research Centre Grow Up Well, The University of Newcastle, Newcastle, NSW Australia; 63 Department of Child and Adolescent Psychiatry, Psychiatric University Hospital Zurich, University of Zurich, Zurich, Switzerland; 64 PROMENTA Research Center, Department of Psychology, University of Oslo, Oslo, Norway; 65 Department of Psychiatry, Sir Run Run Shaw Hospital, School of Medicine, Zhejiang University, Hangzhou, China; 66 Key Laboratory of Medical Neurobiology of Zhejiang Province, School of Medicine, Zhejiang University, Hangzhou, China; 67 Imaging Genetics Center, Mark and Mary Stevens Neuroimaging and Informatics Institute, Keck School of Medicine, University of Southern California, Los Angeles, CA USA; 68 Institute of Neuroscience and Psychology, University of Glasgow, Glasgow, UK; 69 Department of Child and Adolescent Psychiatry, Charité Universitätsmedizin, Berlin, Germany; 70 Early Intervention in Psychosis Advisory Unit for South-East Norway, TIPS Sør-Øst, Division of Mental Health and Addiction, Oslo University Hospital, Oslo, Norway; 71 Department of Psychiatry and Neuropsychology, School for Mental Health and Neuroscience, Faculty of Health Medicine and Life Sciences, Maastricht University, Maastricht, The Netherlands; 72 Center for the Neurobiology of Learning and Memory, University of California Irvine, Irvine, CA USA; 73 Clinical Translational Neuroscience Laboratory, Department of Psychiatry and Human Behavior, University of California Irvine, Irvine, CA USA; 74 Maryland Psychiatric Research Center, University of Maryland School of Medicine, Baltimore, MD USA; 75 Department of Psychology, University of Oslo, Oslo, Norway; 76 School of Psychology, University of Birmingham, Birmingham, UK; 77 Center for Translational Magnetic Resonance Research, Yong Loo Lin School of Medicine, National University of Singapore, Singapore, Singapore; 78 Department of Psychiatry and Behavioral Sciences, Boston Children’s Hospital, Boston, MA USA; 79 Department of Psychiatry, Harvard Medical School, Cambridge, MA USA; 80 OASIS Service, South London and Maudsley NHS Foundation Trust, London, UK; 81 Department of Brain and Behavioural Sciences, University of Pavia, Pavia, Italy; 82 Laboratory of Experimental Psychiatry, Instituto Nacional de Neurología y Neurocirugía, Mexico City, Mexico; 83 Translational Research Center, University Hospital of Psychiatry and Psychotherapy, University of Bern, Bern, Switzerland; 84 Department of Psychological Science, University of California Irvine, Irvine, CA USA; 85 Priority Research Centre for Stroke and Brain Injury, The University of Newcastle, Newcastle, NSW Australia; 86 Faculty of Social Sciences, University of Stavanger, Stavanger, Norway; 87 TIPS Centre for Clinical Research in Psychosis, Stavanger University Hospital, Stavanger, Norway; 88 Clinic for Child and Adolescent Psychiatry, University Hospital of Heidelberg, Heidelberg, Germany; 89 Faculty of Behavioural and Movement Sciences, Department of Clinical, Neuro and Developmental Psychology, Vrije Universiteit Amsterdam, Amsterdam, the Netherlands; 90 National Clinical Research Center for Geriatric Disorders, Xiangya Hospital, Central South University, Changsha, Hunan China; 91 Department of Psychiatry, Psychiatry Neuroimaging Laboratory, Brigham and Women’s Hospital, Harvard Medical School, Boston, MA USA; 92 Department of Psychiatry, Amsterdam University Medical Centre, Amsterdam, the Netherlands; 93 Arkin, Amsterdam, the Netherlands; 94 Emergency Department, Hospital Fray Bernardino Álvarez, Mexico City, Mexico; 95 Tokyo Metropolitan Matsuzawa Hospital, Tokyo, Japan; 96 Hunan Key Laboratory of Psychiatry and Mental Health, the Second Xiangya Hospital, Central South University, Changsha, Hunan China; 97 Magnetic Resonance Imaging Core Facility, Institut d’Investigacions Biomèdiques August Pi i Sunyer, Barcelona, Spain; 98 Neuropsychiatry, The Royal Melbourne Hospital, Melbourne, VIC Australia; 99 Department of Psychiatry & Behavioral Sciences, University of Minnesota, Minneapolis, MN USA; 100 Department of Psychiatry, Hamamatsu University School of Medicine, Hamamatsu, Japan

**Keywords:** Neuroscience, Molecular neuroscience

## Abstract

Individuals at Clinical High Risk for Psychosis (CHR-P) demonstrate heterogeneity in clinical profiles and outcome features. However, the extent of neuroanatomical heterogeneity in the CHR-P state is largely undetermined. We aimed to quantify the neuroanatomical heterogeneity in structural magnetic resonance imaging measures of cortical surface area (SA), cortical thickness (CT), subcortical volume (SV), and intracranial volume (ICV) in CHR-P individuals compared with healthy controls (HC), and in relation to subsequent transition to a first episode of psychosis. The ENIGMA CHR-P consortium applied a harmonised analysis to neuroimaging data across 29 international sites, including 1579 CHR-P individuals and 1243 HC, offering the largest pooled CHR-P neuroimaging dataset to date. Regional heterogeneity was indexed with the Variability Ratio (VR) and Coefficient of Variation (CV) ratio applied at the group level. Personalised estimates of heterogeneity of SA, CT and SV brain profiles were indexed with the novel Person-Based Similarity Index (PBSI), with two complementary applications. First, to assess the extent of within-diagnosis similarity or divergence of neuroanatomical profiles between individuals. Second, using a normative modelling approach, to assess the ‘normativeness’ of neuroanatomical profiles in individuals at CHR-P. CHR-P individuals demonstrated no greater regional heterogeneity after applying FDR corrections. However, PBSI scores indicated significantly greater neuroanatomical divergence in global SA, CT and SV profiles in CHR-P individuals compared with HC. Normative PBSI analysis identified 11 CHR-P individuals (0.70%) with marked deviation (>1.5 SD) in SA, 118 (7.47%) in CT and 161 (10.20%) in SV. Psychosis transition was not significantly associated with any measure of heterogeneity. Overall, our examination of neuroanatomical heterogeneity within the CHR-P state indicated greater divergence in neuroanatomical profiles at an individual level, irrespective of psychosis conversion. Further large-scale investigations are required of those who demonstrate marked deviation.

## Introduction

The Clinical High-Risk state for Psychosis (CHR-P) [1] describes individuals who are at an increased risk of later developing psychosis and can benefit from early intervention, usually implemented in specialised clinics that are emerging worldwide [2, 3]. Individuals at CHR-P accumulate various risk factors for psychosis [4, 5] and have about 50-fold increased risk of transitioning to a first episode of psychosis (FEP) compared to healthy controls (HC) [6]. The CHR-P state consists of several subgroups, each with varying clinical profiles: Attenuated Psychotic Symptoms (APS), Brief Limited Intermittent Psychotic Symptoms (BLIPS) and/or genetic vulnerability accompanied by a deterioration in functioning (GRD) [7–9]. Furthermore, individuals at CHR-P have a highly variable risk enrichment [10] and substantial clinical heterogeneity in initial symptoms, functional status, transition to psychosis, and remission or persistence of symptoms [11–16]. In fact, this observed heterogeneity in clinical and outcome features has been a source of ongoing criticism of the CHR-P paradigm [17, 18]. Such heterogeneity poses a challenge to determining treatment responsivity and the prediction of longitudinal outcomes.

Substantial research efforts have focused on the identification of neuroanatomical abnormalities in individuals at CHR-P, investigated with structural magnetic resonance imaging (sMRI) [19–24]. For example, the Enhancing NeuroImaging Genetics through Meta-Analysis (ENIGMA) [25] consortium recently established the CHR-P Working Group [20] offering the largest pooled structural neuroimaging CHR-P dataset to date. The working group identified widespread deficits in cortical thickness in those at CHR-P compared with HC, which was associated with a transition to psychosis [20]. As such, there have been similar efforts to harness the findings of neuroanatomical deficits to improve the detection of cases and the prediction of transition to FEP [26–28]. However, to date, no reliable neuroanatomical biomarkers have been established, raising the hypothesis of underlying heterogeneity in MRI-based estimates of morphometry and associated neurobiological profiles within the CHR-P state [29, 30].

Emerging statistical measures have made it easier to investigate group-level or personalised estimates of variability in neuroanatomical measures. Heterogeneity within specific anatomical regions can be quantified using the Variability Ratio (VR) or Coefficient of Variation (CV) ratio [31], which have been used to demonstrate greater group-level variability (i.e. heterogeneity) in volumetric measures of the putamen, temporal lobe, thalamus and third ventricle, and lower variability (i.e. homogeneity) in the anterior cingulate cortex of patients with schizophrenia compared to HC [32]. Furthermore, a recent meta-analysis that investigated variability across a narrow subset of structural volumetric brain regions, indexed with the VR, reported no significant differences between individuals at CHR-P and HC, or between those who subsequently transitioned to psychosis and those who did not [33]. Taken together, these findings suggest that variability, as measured by VR, is not significantly different in CHR-P vs. HC.

However, these results stand in contrast to studies that use alternative indices of variability. The Person-Based Similarity Index (PBSI) yields a personalised metric representing inter-subject correlations of neuroanatomical profiles [34–36], and has received recent attention in the context of psychiatric samples, including individuals with bipolar disorder [35, 36] and schizophrenia [36]. The PBSI was recently compared between CHR-P (*n* = 71), FEP (*n* = 72) and HC (*n* = 55) [37], revealing heterogeneity at a personalised level in CHR-P samples. Further, those demonstrating the most marked deviation also demonstrated generally lower IQ and poorer psychopathology [37]. These findings are in contrast with the former meta-analytic findings [33]. However, these incongruities may be explained by the discrepant indices applied, the narrow focus of the brain regions studied meta-analytically [33] and/or the relatively small sample recruited for the PBSI investigations [37]. Taken together, the existing literature offers an ambiguous picture of neuroanatomical heterogeneity in the CHR-P state; as such, further investigations are warranted.

The rationale for elucidating neuroanatomical heterogeneity in the context of CHR is four-fold. First, by examining neuroanatomical heterogeneity in CHR-P, we will gain a fuller understanding of neuroanatomy of the CHR-P population, which allows us to better address criticisms of the CHR-P paradigm which often centre around heterogeneity. Then, this increased understanding may inform the development of precision and predictive models of psychosis. Third, modelling neuroanatomical heterogeneity offers a unique opportunity to identify individuals with potentially shared characteristics of importance. Finally, through subgroup investigations stratified by clinical features, such as a transition to psychosis status and subgroup status (i.e. APS/BLIPS/GRD), we could identify clinical relevance associated with neuroanatomical heterogeneity.

The ENIGMA [25] consortium offers rich structural neuroimaging data across a diverse sample at CHR-P [20], and therefore presents a unique opportunity to systematically address the issue of heterogeneity in this population. Here, we aimed to apply both group-level and personalised indices to investigate whether neuroanatomical heterogeneity differed significantly between; (i) individuals at CHR-P and HC, and (ii) individuals at CHR-P who subsequently transitioned to psychosis and those who did not. In line with the widely reported significant differences between CHR-P and HC in mean neuroanatomical measures, we hypothesised that variance will also significantly differ between the two groups. This assumption is directed by the observation of heightened heterogeneity in other aspects of the CHR-P paradigm, the current lack of successful biomarkers in the CHR-P field and the corresponding potential for discrepant underpinning neurobiological processes. Specifically, we hypothesised that individuals at CHR-P will demonstrate significantly increased heterogeneity in neuroanatomical measures, as demonstrated by significantly higher VR effect sizes and significantly lower PBSI scores.

## Methods

This study was conducted according to the Reporting of studies Conducted using Observational Routinely-collected health Data (RECORD) Statement [38] (eTable 1).

### Participants

The ENIGMA CHR-P dataset amalgamated clinical and neuroimaging data from 29 sites, comprising 1579 individuals meeting CHR-P criteria (according to Comprehensive Assessment of At-Risk Mental States [CAARMS] [9] or the Structured Interview for Prodromal Syndromes [SIPS] [39, 40]) and 1243 HC participants. Longitudinal clinical data that measured transition to psychosis, were also recorded (transition rate [*n* = 226, 14.31%], follow-up duration in months [mean = 28.07, SD = 32.50]). Each site obtained ethics committee approval prior to data collection, and participants provided informed consent or assent prior to participation. Further participant inclusion and exclusion criteria have been previously described [20], and sample discrepancies with the original ENIGMA CHR-P study are detailed in eFig. 1.

### MRI data acquisition and processing

The site-specific MRI acquisition parameters are summarised in eTable 2. All neuroimaging data were processed according to FreeSurfer automated pipelines [41–44] and the standardised ENIGMA protocol (http://enigma.ini.usc.edu/protocols/imaging-protocols/). Briefly, the FreeSurfer pipeline includes motion correction, automated Talairach transformation [45], skull stripping [46], segmentation of the subcortical white matter and grey matter volumetric structures [43, 47], and intensity normalisation [48]. The ENIGMA quality control procedure identifies outliers (±2 SD from the mean) and includes a visual inspection of all images to remove poorly segmented regions, thus resulting in minor fluctuation in sample size for each region of interest (ROI). The application of this protocol yielded a total of 153 structural ROIs: 68 cortical variables measured by both Surface Area (SA) and Cortical Thickness (CT) according to the Desikan–Killiany atlas [49], 16 Subcortical Volume (SV) variables and one measure of Intracranial Volume (ICV). Participants with >5% missing ROIs were excluded from the current analyses as this was deemed to be indicative of poor parcellation (eFig. 1).

Neuroimaging data were adjusted for scanner protocol and site using neuroComBat [50] (a modified version of ComBat [51]), a batch-adjustment method that relies on an empirical Bayes framework to assess the influence of covariates of interest. The neuroimaging data were adjusted prior to current analyses, as this approach is recommended by the tool developers for optimal use while controlling for group (CHR-P/HC), age and sex. NeuroComBat has previously been validated on data derived from the ENIGMA protocol described above (in the ENIGMA SCZ dataset) [52] and allows for partially missing data [50]. In previous work using this dataset, we have empirically demonstrated that applying neuroComBat to the data reported here leads to more precise estimates of effect sizes, both compared to non-neuroComBat-corrected data and random-effects meta-analysis [20].

### Statistical analysis

All analyses were conducted within *R v*.4.0.3 [53]; the VR analyses were conducted using the *metafor* [54] and *meta* [55] packages. Effect sizes were previously reported for group differences in each ROI between CHR-P/HC and transition status [20]; as such, the current analysis provides an in-depth exploration of neuroanatomical heterogeneity in this dataset using baseline clinical and neuroimaging data and longitudinal clinical outcome data.

#### Variability ratio and coefficient of variation

We applied the log-VR using the escalc() function; this statistical index has gained recent attention as an indicator of inter-individual variability for various clinical factors, such as treatment effect [31, 56], and is calculated according to the formula below:

$${\rm{In}}\,{\rm{VR}} = {\rm{In}}\left( {\frac{{\hat \sigma _p}}{{\hat \sigma _c}}} \right) = {\rm{In}}\left( {\frac{{S_p}}{{S_c}}} \right) + \frac{1}{{2\left( {n_p - 1} \right)}} - \frac{1}{{2\left( {n_c - 1} \right)}}$$

where $\hat \sigma _p$ and $\hat \sigma _c$ are the unbiased estimates of population SDs; *S*_*p*_ and *S*_*c*_ are the reported sample SDs; *n*_*p*_ and *n*_*c*_ are the sample sizes for CHR-P (or CHR-T/APS) and HC (or CHR-NT) groups, respectively.

This calculation was conducted across each ROI to compare baseline variability in regional neuroanatomical measures between CHR-P and HC in the first instance, and then between CHR-P individuals who transitioned to FEP (CHR-T) and those who did not (CHR-NT). CHR-P participants who were lost to follow-up (*n* = 258) were not included in the latter investigation (eFig. 1). We also conducted further exploratory applications limited to those meeting APS subgroup criteria compared with HC. Due to the low prevalence of the BLIPS and GRD subgroups (see Table 1) and the corresponding high volume of ROIs under investigation, it was not feasible to conduct analyses limited to these two subgroups, respectively.

**Table 1:** Sample characteristics for the clinical-high risk for psychosis (CHR-P) and the healthy control (HC) groups.

	CHR-P (*N* = 1579)	HC (*N* = 1243)
Age in years, mean (SD)	20.63 (4.60)	22.32 (4.96)
Sex, M/F	831/748	687/556
Transition to psychosis, %	14.31	NA
Follow-up duration in months, mean (SD)	28.07 (32.50)	NA
Typical antipsychotics, *n* (%)	15 (0.95%)	NA
Atypical antipsychotics, *n* (%)	216 (13.68%)	NA
Total severity symptoms score^a^, mean (SD)	CAARMS: 10.34 (4.03) SIPS: 10.93 (4.66)	NA
Subgroups^b^, *n* (%)	APS: 1177 (74.54%)	NA
	BLIPS: 46 (2.91%)	
	GRD: 90 (5.70%)	
	APS/GRD: 129 (8.17%)	
	APS/BLIPS: 27 (1.71%)	
	BLIPS/GRD: 2 (0.13%%)	
	APS/BLIPS/GRD: 7 (0.44%)	
	Unknown: 101 (6.40%%)	

^a^243 participants had neither the CAARMS nor SIPS assessment scores provided.

^b^*APS* Attenuated Psychotic Symptoms; *BLIPS* Brief Limited Intermittent Psychotic Symptoms; *GRD* Genetic and Risk Deterioration Syndrome; some participants met criteria for more than one subgroup.

The log-VR was back-transformed into linear scale (VR) to aid interpretation of the results. Therefore, a VR of 1 indicates equal variability in neuroanatomical measures between groups. A VR > 1 suggests greater variability in the CHR-P group (or CHR-T and APS, respectively), whereas a VR < 1 indicates less variability in the CHR-P group. The VR (with 95% confidence intervals) for each ROI were then summarised in forest plots according to SA, CT, SV and ICV. Given the high number of ROI tests conducted, we calculated *p* value adjustments using the False Discovery Rate (FDR) [57] approach, applied to all of the ROIs as one vector at once. As such, the forest plots report both the uncorrected and corrected *p*-values.

Previous research within the ENIGMA CHR dataset identified between-group mean differences of sMRI measures [20]. As the log variability ratio (log-VR) is not scaled to the mean, we conducted a supplementary calculation of the log Coefficient of Variation (log-CV) ratio index, which offers a mean-scaled metric of variability between two groups and is calculated according to the formula below [31]. In instances in which the CHR-P population (or CHR-T/APS groups) demonstrate lower mean sMRI values compared with the HC population (or CHR-NT), the log-VR offers the more conservative test of our hypotheses. However, in instances of larger mean values in the CHR-P population or the transition to psychosis group, the log-CV offers the more conservative test. As previous research in this data set largely described lower mean values across sMRI measures in the CHR-P population, particularly regarding measures of CT [20], we calculated the log-CV to supplement the findings of the primary log-VR analyses.

$${\rm{In}}\,{\rm{CVR}} = {\rm{In}}\left( {\frac{{\hat \sigma _p/\bar x_p}}{{\hat \sigma _c/\bar x_c}}} \right) = {\rm{In}}\left( {\frac{{S_p/\bar x_p}}{{S_c/\bar x_c}}} \right) + \frac{1}{{2\left( {n_p - 1} \right)}} - \frac{1}{{2\left( {n_c - 1} \right)}}$$

where $\bar x_p$ and $\bar x_c$ are the reported means for the CHR-P (or CHR-T/APS) and HC (or CHR-NT) groups.

Finally, we conducted sensitivity analyses on ROIs demonstrating significant effects in the primary analyses, to better elucidate whether identified effects might be better explained in part by factors associated with suboptimal study design as opposed to meaningful neurobiological mechanisms. These analyses included leave-one-out resampling to investigate site effects (eMethods 1), and supplementary testing on an age-, sex-, and site-matched sample (eMethods 2) to control for other potential sources of heterogeneity.

#### Person-Based Similarity Index

The personalised estimates of inter-individual variability were investigated using the PBSI, calculated according to the formula below, for each SA, CT and SV profiles [34–37]. The process for calculating the PBSI scores begins with concatenating the respective regional measures into vectors that represent the profile of each specific brain phenotype; PBSI-SA, PBSI-CT and PBSI-SV, respectively. This produces a simplified, personalised index for each phenotypic neuroanatomical profile. This index can then be used in one of two ways; first, to quantify how similar an individuals’ brain profile is to that of other individuals with the same clinical profile or disorder (within-diagnosis or within-group). Second, to quantify how similar an individuals’ brain profile is respective to a normative estimate, i.e. the average of the healthy control group (normativeness) [37].

$${\rm{PBSI}}_i = \frac{1}{{N - 1}}\mathop {\sum}\limits_{j \ne i} {{\rm{cor}}\left( {y_i,\,y_j} \right)}$$

The PBSI of the *i*th individual is the average correlation between his/her brain measures (*y*_*i*_) and the brain measures of any other individual of the reference sample (*y*_*j*_, for *j* ≠ *i*).

(i) *Within-group reference:* The PBSI-SA, PBSI-CT and PBSI-SV were calculated separately for the CHR-P and HC individuals and thus represent the degree of within-group similarity in these profiles. Within each group, and for each brain phenotype, Spearman correlation coefficients were computed between the neuroanatomical profile of each participant and the profiles of each other member of the same group. The average of these coefficients for each participant yielded their respective PBSI score for each brain phenotype. A higher PBSI score (closer to 1) indicates greater similarity in the neuroanatomical profile of an individual to other members of the same group, while a lower score indicates greater deviance in their neuroanatomical profile. Group-level comparisons of PBSI-SA, PBSI-CT and PBSI-SV were then conducted between CHR-P and HC using one-tailed Welch’s *t* tests to examine whether psychosis-risk states were associated with greater within-group variability.
(ii) *Normative reference:* Next, the respective neuroanatomical profiles of each CHR-P individual were correlated with the corresponding profiles of the members of the HC group, for each brain phenotype. The resulting PBSI scores thus represent the degree of deviation from the ‘normative’ range and were transformed into *z*-scores (PBSI-CT-Z, PBSI-SA-Z, and PBSI-SV-Z). We set >1.5 SD as a threshold to identify individuals at CHR-P who most markedly deviated from the normative neuroanatomical profile, in line with previous work [37].

In both PBSI analyses, we also investigated the potentially moderating effects of transition status (CHR-T/CHR-NT), subgroup status (APS/BLIPS/GRD), antipsychotic exposure, and overall baseline psychopathology (total CAARMS/SIPS severity *z*-scores, eMethods 3) on PBSI scores. All multivariable regression models were applied, adjusting for age and sex.

## Results

### Sample characteristics

Following quality control procedures (eFig. 1), the final sample consisted of 1 579 CHR-P participants (mean age = 20.63 [SD = 4.60], 47.37% females) and 1243 HC participants (mean age = 22.32 [SD = 4.96], 44.73% females) across 29 sites. Table 1 provides a detailed sample summary. Of the CHR-P participants, 1248 also had longitudinal clinical data; the length of follow-up ranged from 1 to 194 months (mean = 28.07 [SD = 32.50], median = 18.00). eTable 3 provides a detailed comparison of the CHR-T and CHR-NT groups.

### Variability ratio and coefficient of variation

#### CHR-P compared with HC

##### Regional SA

**Fig. 1:**
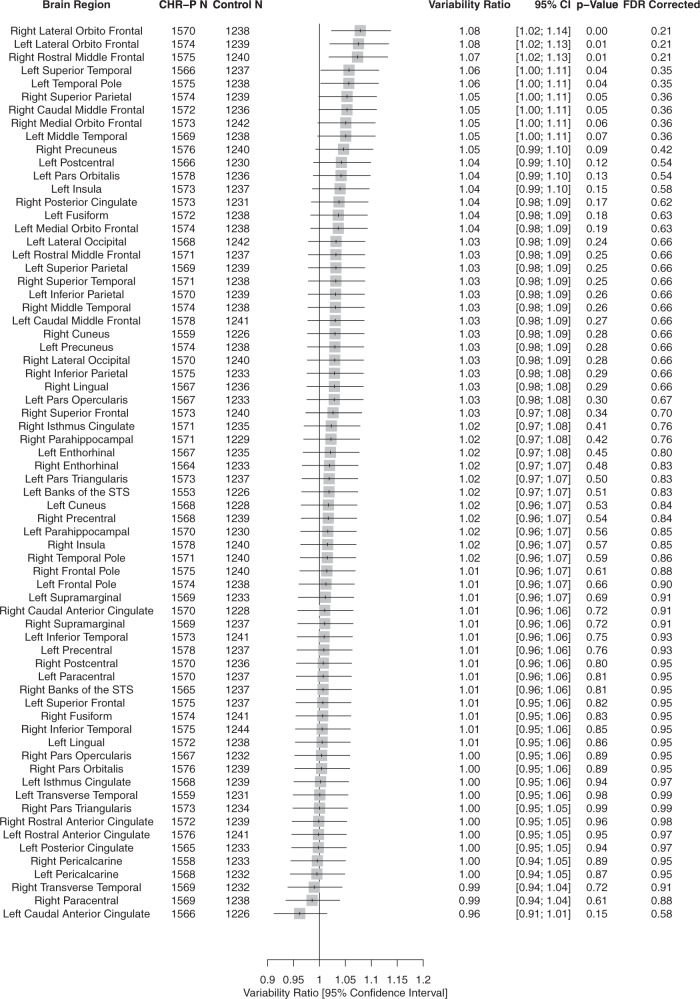
Forest plot of the variability ratio (VR) of cortical surface area (SA) measures in CHR-P compared with healthy controls. CHR-P clinical high risk for psychosis, STS superior temporal sulcus, VR variability ratio.

While the CHR-P group demonstrated a trend towards greater variability compared to the HC group in measures of cortical SA in the right lateral orbitofrontal region (VR = 1.08, 95% CI: 1.02–1.14), left lateral orbitofrontal region (VR = 1.08, 95% CI: 1.02–1.13) and right rostral middle-frontal region (VR = 1.07, 95% CI: 1.02–1.13), these observations did not survive FDR adjustments. No SA regions demonstrated significantly greater homogeneity in CHR-P (Fig. 1). These trends were confirmed in CV analyses (eFig. 2).

##### Regional CT

**Fig. 2:**
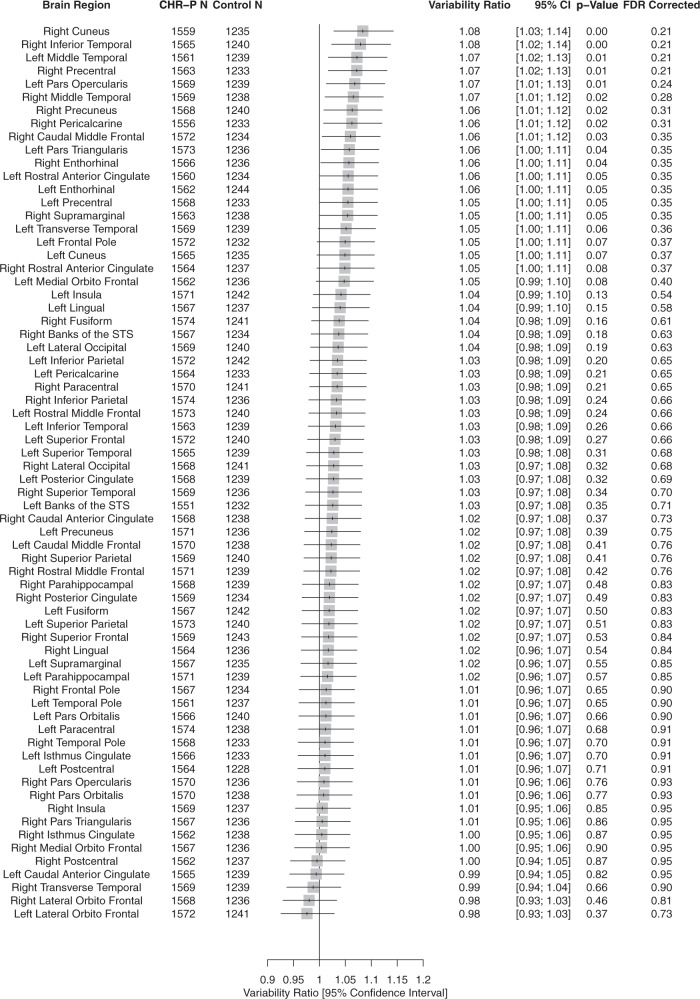
Forest plot of the variability ratio (VR) of cortical thickness (CT) measures in CHR-P compared with healthy controls. CHR-P clinical high risk for psychosis, STS superior temporal sulcus, VR variability ratio.

There was a trend towards greater heterogeneity in CHR-P compared to HC in the right cuneus (VR = 1.08, 95% CI:1.03–1.14), right inferior-temporal region (VR = 1.08, 95% CI:1.02–1.14), left middle-temporal region (VR = 1.07, 95% CI:1.02–1.13), right precentral region (VR = 1.07, 95% CI:1.00–1.15, *p* = 0.01) and left pars opercularis (VR = 1.07, 95% CI: 1.02–1.13). Again, these observations did not remain statistically significant after applying FDR corrections. No regions demonstrated greater homogeneity in CHR-P compared to HC (Fig. 2). Supplementary CV analyses (eFig. 3) supported these findings.

##### Regional SV

**Fig. 3:**
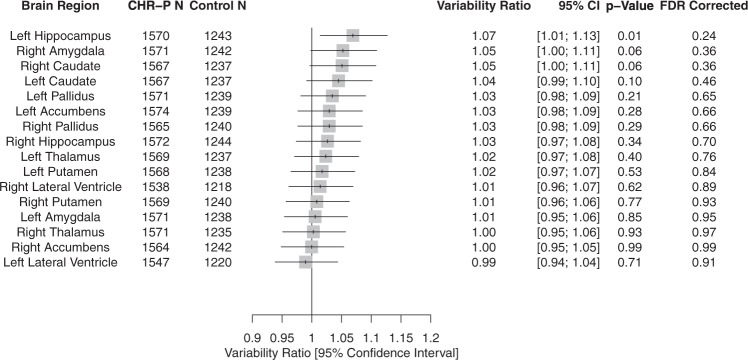
Forest plot of the variability ratio (VR) of subcortical volume (SV) measures in CHR-P compared with healthy controls. CHR-P clinical high risk for psychosis, VR variability ratio.

There was a numerical trend towards higher heterogeneity in CHR-P compared to HC individuals in the left hippocampus (VR = 1.07, 95% CI: 1.01–1.13), notwithstanding FDR corrections (Fig. 3). Supplementary CV (eFig. 4) analyses corroborated these findings.

##### ICV

No significant differences in ICV heterogeneity or homogeneity were observed between CHR-P and HC, indexed with either the VR (eFig. 5) or CV (eFigu. 6).

#### CHR-T compared with CHR-NT

CHR-P individuals who transitioned to psychosis did not demonstrate significantly greater heterogeneity or homogeneity in regional neuroanatomical measures compared with individuals who did not transition to psychosis, as indexed by both the VR and CV (eFigs. 7–14).

#### APS compared with HC

Individuals meeting the criteria for the APS subtype demonstrated a trend towards greater SA heterogeneity in the left lateral orbitofrontal region (VR = 1.07, 95% CI: 1.01–1.14) compared with HC (eFig. 15), but no regions survived FDR correction for multiple comparisons. No other significant regions were identified in VR or CV analyses (eFigs. 16–22).

### Person-Based Similarity Index

#### Within-group PBSI

**Fig. 4:**
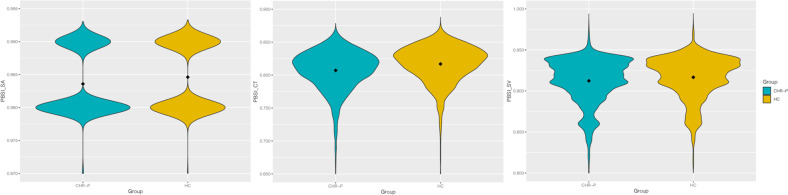
Violin plots comparing the distribution of PBSI scores between individuals at CHR-P and healthy controls, across surface area (PBSI_SA), cortical thickness (PBSI_CT), and subcortical volume (PBSI_SV); the mid-point indicates the group mean. PBSI Person-Based Similarity Index, SA surface area, CT cortical thickness, SV subcortical volume. All three phenotypes demonstrate significantly lower similarity in PBSI profiles in the CHR-P group compared with healthy controls, across PBSI_SA (*p* < 0.01), PBSI_CT (*p* < 0.01), PBSI_SV (*p* < 0.01).

There was greater within-group variability in all neuroanatomical profiles in the CHR-P group compared to the HC group based on significantly lower PBSI-SA (*t*(2642) = −5.39, *p* < 0.01), PBSI-CT (*t*(2788) = −9.11, *p* < 0.01), and PBSI-SV scores (*t*(2733) = −4.34, *p* < 0.01) (Fig. 4). PBSI-CT scores were substantially lower than PBSI-SA and PBSI-SV (Fig. 4), signalling greater divergence specifically in CT profiles. There were no significant associations between PBSI scores and transition or subgroup status, baseline psychopathology (all *p* > 0.12), or current typical or atypical antipsychotic use on PBSI-SA or PBSI-CT scores. There was a slight association of typical antipsychotic use with PBSI-SV scores, albeit not surviving the stricter significance threshold (*b* = −0.02, *t*(1220) = −2.017, *p* = 0.04).

#### Normative PBSI

**Fig. 5:**
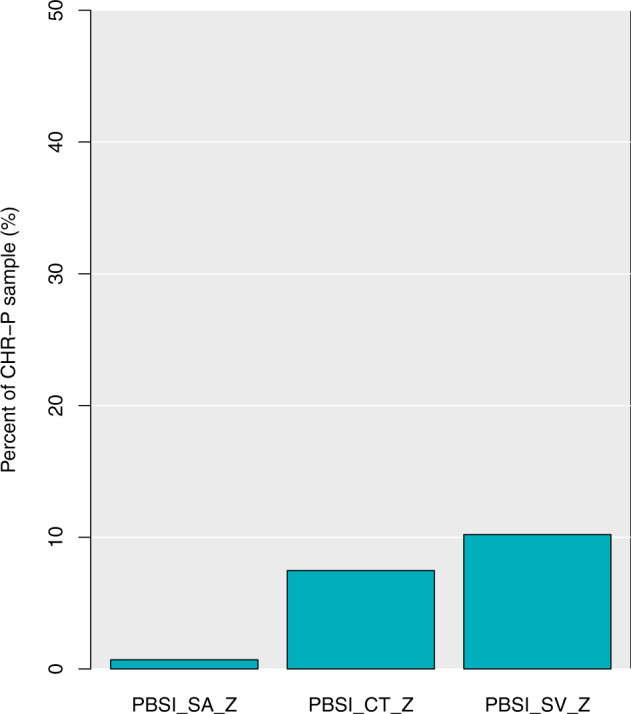
A bar chart representing the percentage of the CHR-P sample who demonstrate marked deviation from the ‘normative’ neuroanatomical profile. CHR-P clinical high risk for psychosis, PBSI_SA_Z person-based similarity index surface area z-scores, PBSI_CT_Z person-based similarity index cortical thickness z-scores, PBSI_SV_Z person-based similarity index subcortical volume z-scores.

Of the 1579 CHR-P participants, 11 (0.70%) demonstrated marked deviation in PBSI-SA-Z scores, 118 (7.47%) in PBSI-CT-Z and 161 (10.20%) in PBSI-SV-Z (Fig. 5). Of these participants, 17 demonstrated marked deviation in more than one phenotypic profile and just one participant in all three phenotypic profiles. There were no significant associations between normative PBSI scores and transition or subgroup status, or baseline psychopathology (all *p* > 0.18). A significant association with typical antipsychotic use was identified for the PBSI-SV-Z scores (*b* = −0.84, *t*(1220) = −2.191, *p* = 0.03), with antipsychotic use being associated with greater deviations from PSBI-SV-Z. No association with antipsychotic use was identified with PBSI-SA-Z or PBIS-CT-Z scores.

## Discussion

We conducted a large-scale investigation of neuroanatomical heterogeneity in a help-seeking population meeting CHR-P criteria. To summarise, we observed a trend towards regional heterogeneity (as measured by the variability ratio) in a cluster of frontal, temporal and hippocampal regions that failed to reach statistical significance after correction for multiple comparisons. However, Person-Based Similarity Index (PBSI) analyses, a novel measure of inter-individual variability, indicated greater divergence in global neuroanatomical profiles of SA, CT and SV in CHR-P compared with HC. Importantly, however, the proportion of CHR-P individuals with significantly deviant PBSI scores was low. Moreover, none of the variability metrics examined showed significant associations with a transition to psychosis.

Our first key finding was an observed trend towards heightened heterogeneity in individuals at CHR-P in a cluster of frontal, temporal and hippocampal regions compared with HC. This result is in line with the fine-grained and localised alterations typically observed in the CHR-P state. Existing literature has identified structural, [26, 58–60] functional [26, 60], and neurocognitive [61] alterations in frontal and medial-frontal regions in the CHR-P state, [26, 58–60] and further highlighted these as potentially important regions in the pathophysiology of psychosis. [26, 58–60] Similarly, aberrations in temporal [26, 59, 60, 62] and hippocampal regions [26, 59, 63–66] have also been identified in CHR-P and have been implicated as core regions in the transition to psychosis. To observe localised heterogeneity in these regions might signal discrepant neurobiological processes associated with psychosis-risk states (or with psychosis conversion in subsequent CHR-T/CHR-NT analyses), which may ultimately prove useful for stratification purposes in interventional research. However, all observed effect sizes were small (1.06–1.08) and these findings did not survive the FDR correction for multiple comparisons. Furthermore, no significant effects of the transition to psychosis were identified. These results are consistent with a recent meta-analysis that applied the VR across a smaller subset of volumetric regions and similarly identified no significant regions of increased variability in CHR-P [33]. Equally, a previous study that compared CHR-P (*n* = 71) and HC (*n* = 55), indexed with the CV metric, found no evidence of regional increases in variability in CHR-P [37], demonstrating the robustness of these findings. Taken together, these findings, in combination with ours, suggest that regional neuroanatomical variability in the CHR-P state is not significantly different from healthy controls.

However, the application of the PBSI offered a somewhat contrasting conclusion. The within-diagnosis PBSI estimates revealed significantly lower scores across global SA, CT and SV amongst individuals at CHR-P, compared with HC. These findings signal greater divergence in neuroanatomical profiles within the CHR-P state across all three phenotypic measures. This finding is largely consistent with previous research which identified lower PBSI-CT and PBSI-SV scores in individuals at CHR-P compared with HC [37]. Notably, higher variability in CT profiles was also reported in another sample of patients with schizophrenia compared to HC [36]. These findings suggest that higher inter-individual variability in cortical and subcortical phenotypes is a consistent feature both at the at-risk stage and after the onset of FEP. This is also particularly interesting within the context of previous findings in the ENIGMA CHR-P dataset of widespread CT deficits [20], and warrants further investigation of variance specifically in CT phenotypes across the psychosis spectrum.

Crucially, normative modelling of the PBSI also identified a sub-sample of CHR-P individuals who demonstrated marked deviation in reference to a ‘normative’ neuroanatomical profile. The identification of deviations from normative modelling is becoming increasingly popular in psychiatry and may aid in the classification of distinct subgroups. [35–37, 67] Although <1% of the sample displayed markedly ‘deviant’ PBSI-SA scores, this rose to 7.47% for PBSI-CT and 10.20% for PBSI-SV scores, suggesting that approximately 7–10 out of 100 CHR-P individuals have markedly deviant neuroanatomical profiles in SV or CT compared to HC. Together, the PBSI findings indicate the potential utility of examining personalised indexes as opposed to employing group-level estimations of variance. However, the observed heterogeneity in CHR-P individuals was not significantly associated with severity of baseline attenuated psychotic psychopathology, subgroup allocation (APS/BLIPS/GRD) or transition to psychosis. These findings suggest that neuroanatomical variability is not linked to the clinical features we examined.

The lack of an association between heterogeneity and transition to psychosis may reflect the challenges we face when employing dichotomous diagnostic criteria—particularly as psychosis risk is associated with various transdiagnostic outcomes [16]. At this time, we were unable to assess the link between neuroanatomical heterogeneity and other longitudinal clinical outcomes, such as psychosocial functioning, non-psychotic psychopathology or persistence of attenuated symptoms. However, harmonisation of additional outcome measures is an ongoing endeavour of the ENIGMA CHR working group; therefore, in the future, we plan to examine how neuroanatomical heterogeneity is associated with other measures. Given the prevalence and variability of these alternative outcomes in the CHR-P state [68–70], it will be important to assess whether these hold greater associations with neuroanatomical variability in order to better address the clinical relevance of neuroanatomical heterogeneity. In this respect, it may be especially pertinent to investigate the subgroup of individuals at CHR-P who markedly deviated from the ‘norm’ in the PBSI analyses. Furthermore, there was substantial variation in follow-up duration between sites. As such, it is possible that the presence of individuals at CHR-P that were classified as ‘no transition’—yet who may have developed psychosis following their final data contributions—may have reduced our power to detect group differences.

There are also further methodological limitations to consider. First, the validity of the VR as an index of heterogeneity has been debated, particularly within the context of other clinical factors, such as individual treatment response and subgroup effects [71]. While we performed additional individual-level PBSI analyses to supplement the VR analyses, the indices produced somewhat conceptually discrepant findings. These discrepancies may be underpinned by the group-level approach of the VR index as opposed to the individual-level PBSI scores, or alternatively due to the nature of the PBSI scores which capture overall patterns of neuroanatomical heterogeneity as opposed to specific regional patterns. It is possible that adopting a global approach offers a more powerful examination of heterogeneity compared to a region-by-region approach. Nevertheless, these current findings corroborate existing literature which reported significant differences in variability of neuroanatomical profiles with the application of PBSI scores [37], and a lack thereof with a regional group-level VR [33] or CV [37] approach. However, the current findings also necessitate further validation and critical appraisal of the various indices of heterogeneity. Heterogeneity has recently become a mainstay focus of clinical research—particularly in psychiatry—and it is imperative to systematically compare the statistical performance of the relevant indices in order to develop a gold standard framework for addressing questions of variance.

Second, we were also unable to control for further potentially confounding factors, such as substance use. Given the potential impact of alcohol, tobacco and cannabis use on neuroanatomical profiles in CHR-P [72, 73], it will be important to assess these features as this consortium continues to develop and expand. Future research should also continue to explore heterogeneity within the CHR-P paradigm, both within neurobiological bases and other characteristics. The elucidation of such sources of heterogeneity will be essential in order to improve prognostic research paradigms in this population [74].

### Future directions

Given these limitations, there is a range of next steps to further elucidate neuroanatomical heterogeneity in the CHR-P paradigm. First, as the ENIGMA CHR-P Working Group continues to develop and expand, it would be interesting to incorporate genomic data to assess the genetic contributions to population variability in neuroimaging phenotypes, such as the schizophrenia polygenic risk score [75], as well as assessing the association of neuroanatomical heterogeneity with alternative clinical and functional outcomes outside of transition to psychosis. Finally, once longitudinal neuroimaging data becomes available, it will also be important to assess the longitudinal stability of the neuroanatomical heterogeneity findings here.

## Conclusions

In the largest pooled neuroimaging sample of individuals at CHR-P to date, we identified an absence of significantly greater regional heterogeneity compared with HC, despite an emerging trend towards greater fronto-temporal and hippocampal heterogeneity in CHR-P. These findings persist irrespective of longitudinal transition to psychosis. Subsequent application of a personalised PBSI score revealed significantly greater divergence in global neuroanatomical profiles in CHR-P, and further, a small subgroup (approximately 10%) of individuals at CHR-P who demonstrate markedly divergent neuroanatomical profiles of SA, CT and SV respective to a normative profile. Further clinical investigation of this subgroup is required in light of the limited clinical variables currently available.

## Supplementary information

Supplementary Materials

Supplementary Materials - Collaborator list

## Data Availability

Computer code to calculate the VR, CVR and PBSI statistics is available from the authors upon request.
