# Supplementary material for: Neuroanatomical heterogeneity and homogeneity in individuals at clinical high risk for psychosis
Source: Transl Psychiatry. 2022 Jul 26;12:297. doi: 10.1038/s41398-022-02057-y (PMC9325730; doi:10.1038/s41398-022-02057-y)
Supplement: Supplementary file 1 — Supplementary Materials [file 41398_2022_2057_MOESM1_ESM.docx]

***Electronic supplementary materials***

**Abbreviations**

**eTable 1:** The RECORD checklist of items and where they are reported in the manuscript.

**eTable 2:** Structural Magnetic Resonance Imaging scanning parameters for each site.

**eMethods 1:** Leave-one-site-out resampling method.

**eMethods 2.** Age-, sex- and site-matched analyses.

**eMethods 3:** Z-scores indexing the baseline summed severity of symptoms on the CAARMS and SIPS assessments.

**eFigure 1:** Flow diagram to represent the included/excluded participants and the final sample, according to a) individuals at CHR-P, and b) control participants.

**eTable 3:** Sample characteristics for the CHR-P-Transition (CHR-T) and CHR-P-No Transition (CHR-NT) groups.

**eFigure 2:** Forest plot of the Coefficient of Variation (CV) ratio of Surface Area (SA) in individuals at CHR-P compared with HC.

**eFigure 3:** Forest plot of the Coefficient of Variation (CV) ratio of Cortical Thickness (CT) in individuals at CHR-P compared with HC.

**eFigure 4:** Forest plot of the Coefficient of Variation (CV) ratio of Subcortical Volume (SV) in individuals at CHR-P compared with HC.

**eFigure 5:** Forest plot of the Variability Ratio (VR) of Intra-Cranial Volume (ICV) in individuals at CHR-P compared with HC.

**eFigure 6:** Forest plot of the Coefficient of Variation (CV) ratio of Intra-Cranial Volume (ICV) in individuals at CHR-P compared with HC.

**eFigure 7:** Forest plot of the Variability Ratio (VR) of cortical Surface Area (SA) in individuals at CHR-P who subsequently transition to psychosis (CHR-T) compared with those who did not (CHR-NT).

**eFigure 8:** Forest plot of the Coefficient of Variation (CV) ratio of cortical Surface Area (SA) in individuals at CHR-P who subsequently transition to psychosis (CHR-T) compared with those who did not (CHR-NT).

**eFigure 9:** Forest plot of the Variability Ratio (VR) of Cortical Thickness (CT) in individuals at CHR-P who subsequently transition to psychosis (CHR-T) compared with those who did not (CHR-NT).

**eFigure 10:** Forest plot of the Coefficient of Variation (CV) ratio of Cortical Thickness (CT) in individuals at CHR-P who subsequently transition to psychosis (CHR-T) compared with those who did not (CHR-NT).

**eFigure11:** Forest plot of the X Variability Ratio (VR) of Subcortical Volume (SV) in individuals at CHR-P who subsequently transition to psychosis (CHR-T) compared with those who did not (CHR-NT).

**eFigure12:** Forest plot of the Coefficient of Variation (CV) ratio of Subcortical Volume (SV) in individuals at CHR-P who subsequently transition to psychosis (CHR-T) compared with those who did not (CHR-NT).

**eFigure13:** Forest plot of the Variability Ratio (VR) of Intra-Cranial Volume (ICV) in individuals at CHR-P who subsequently transition to psychosis (CHR-T) compared with those who did not (CHR-NT).

**eFigure14:** Forest plot of the Coefficient of Variation (CV) ratio of Intra-Cranial Volume (ICV) in individuals at CHR-P who subsequently transition to psychosis (CHR-T) compared with those who did not (CHR-NT).

**eFigure 15:** Forest plot of the Variability Ratio (VR) of cortical Surface Area (SA) in APS-allocated individuals compared with HC.

**eFigure 16:** Forest plot of the Coefficient of Variation (CV) ratio of cortical Surface Area (SA) in APS-allocated individuals compared with HC.

**eFigure 17:** Forest plot of the Variability Ratio (VR) of Cortical Thickness (CT) in APS-allocated individuals compared with HC.

**eFigure 18:** Forest plot of the Coefficient of Variation (CV) ratio of Cortical Thickness (CT) in APS-allocated individuals compared with HC.

**eFigure 19:** Forest plot of the Variability Ratio (VR) of Subcortical Volume (SV) in APS-allocated individuals compared with HC.

**eFigure 20:** Forest plot of the Coefficient of Variation (CV) ratio of Subcortical Volume (SV) in APS-allocated individuals compared with HC.

**eFigure 21:** Forest plot of the Variability Ratio (VR) of Intra-Cranial Volume (ICV) in APS-allocated individuals compared with HC.

**eFigure 22:** Forest plot of the Coefficient of Variation (CV) ratio of Intra-Cranial Volume (ICV) in APS-allocated individuals compared with HC.

**eReferences**

**Abbreviations**

| **ENIGMA** | Enhancing NeuroImaging Genetics through Meta-Analysis |
| --- | --- |
| **CHR-P** | Clinical High Risk for Psychosis |
| **HC** | Healthy Controls |
| **CHR-T** | Clinical High Risk with longitudinal Transition to psychosis |
| **CHR-NT** | Clinical High Risk without longitudinal Transition to psychosis |
| **ROI** | Region of Interest |
| **CAARMS** | Comprehensive Assessment for At-Risk Mental States |
| **SIPS** | Structured Interview for Prodromal Symptoms |
| **VR** | Variability Ratio |
| **CVR** | Coefficient of Variation Ratio |
| **SA** | Surface Area |
| **CT** | Cortical Thickness |
| **SV** | Subcortical Volume |
| **ICV** | Intra-Cranial Volume |
| **FDR** | False Discovery Rate |

**eTable 1.** The RECORD checklist of items and where they are reported in the manuscript.^1^

|  | **Item No.** | **STROBE items** | **Location in manuscript where items are reported** | **RECORD items** | **Location in manuscript where items are reported** |
| --- | --- | --- | --- | --- | --- |
| **Title and abstract** | | | | | |
|  | 1 | (a) Indicate the study’s design with a commonly used term in the title or the abstract (b) Provide in the abstract an informative and balanced summary of what was done and what was found | Abstract | RECORD 1.1: The type of data used should be specified in the title or abstract. When possible, the name of the databases used should be included.  RECORD 1.2: If applicable, the geographic region and timeframe within which the study took place should be reported in the title or abstract.  RECORD 1.3: If linkage between databases was conducted for the study, this should be clearly stated in the title or abstract. | Abstract  Abstract – “international;”  NA |
| **Introduction** | | | | | |
| Background rationale | 2 | Explain the scientific background and rationale for the investigation being reported | Introduction |  |  |
| Objectives | 3 | State specific objectives, including any prespecified hypotheses | Introduction, Methods |  |  |
| **Methods** | | | | | |
| Study Design | 4 | Present key elements of study design early in the paper | Methods |  |  |
| Setting | 5 | Describe the setting, locations, and relevant dates, including periods of recruitment, exposure, follow-up, and data collection | Methods |  |  |
| Participants | 6 | *(a) Cohort study* - Give the eligibility criteria, and the sources and methods of selection of participants. Describe methods of follow-up  *Case-control study* - Give the eligibility criteria, and the sources and methods of case ascertainment and control selection. Give the rationale for the choice of cases and controls  *Cross-sectional study* - Give the eligibility criteria, and the sources and methods of selection of participants  *(b) Cohort study* - For matched studies, give matching criteria and number of exposed and unexposed  *Case-control study* - For matched studies, give matching criteria and the number of controls per case | a) Partial description in Methods and clear reference to first ENIGMA CHR-P paper for more information (Jalbrzikowski et al, 2021)  b) Supplementary matched cohort analyses – eMethods 4  NA | RECORD 6.1: The methods of study population selection (such as codes or algorithms used to identify subjects) should be listed in detail. If this is not possible, an explanation should be provided.  RECORD 6.2: Any validation studies of the codes or algorithms used to select the population should be referenced. If validation was conducted for this study and not published elsewhere, detailed methods and results should be provided.  RECORD 6.3: If the study involved linkage of databases, consider use of a flow diagram or other graphical display to demonstrate the data linkage process, including the number of individuals with linked data at each stage. | NA  NA  NA |
| Variables | 7 | Clearly define all outcomes, exposures, predictors, potential confounders, and effect modifiers. Give diagnostic criteria, if applicable. | Methods, Table 1 and Supplementary Materials | RECORD 7.1: A complete list of codes and algorithms used to classify exposures, outcomes, confounders, and effect modifiers should be provided. If these cannot be reported, an explanation should be provided. | NA |
| Data sources/ measurement | 8 | For each variable of interest, give sources of data and details of methods of assessment (measurement).  Describe comparability of assessment methods if there is more than one group | Methods, and Supplementary Materials |  |  |
| Bias | 9 | Describe any efforts to address potential sources of bias | Methods/Statistical Analysis/ Supplementary methods |  |  |
| Study size | 10 | Explain how the study size was arrived at | NA – predetermined sample size |  |  |
| Quantitative variables | 11 | Explain how quantitative variables were handled in the analyses. If applicable, describe which groupings were chosen, and why | Statistical Analysis/ Supplementary Methods |  |  |
| Statistical methods | 12 | (a) Describe all statistical methods, including those used to control for confounding  (b) Describe any methods used to examine subgroups and interactions  (c) Explain how missing data were addressed  (d) *Cohort study* - If applicable, explain how loss to follow-up was addressed  *Case-control study* - If applicable, explain how matching of cases and controls was addressed  *Cross-sectional study* - If applicable, describe analytical methods taking account of sampling strategy  (e) Describe any sensitivity analyses | a) Statistical Analysis/ Supplementary Methods  b) Statistical Analysis  c) Statistical Analysis  d) Statistical analysis  NA  NA  Supplementary Methods |  |  |
| Data access and cleaning methods |  | .. |  | RECORD 12.1: Authors should describe the extent to which the investigators had access to the database population used to create the study population.  RECORD 12.2: Authors should provide information on the data cleaning methods used in the study. | Methods  Statistical Analysis |
| Linkage |  | .. |  | RECORD 12.3: State whether the study included person-level, institutional-level, or other data linkage across two or more databases. The methods of linkage and methods of linkage quality evaluation should be provided. | NA |
| **Results** | | | | | |
| Participants | 13 | (a) Report the numbers of individuals at each stage of the study (*e.g.*, numbers potentially eligible, examined for eligibility, confirmed eligible, included in the study, completing follow-up, and analysed)  (b) Give reasons for non-participation at each stage.  (c) Consider use of a flow diagram | a) Results and Supplementary Materials (eFigure 1)  b) eFigure 1  c) eFigure 1 | RECORD 13.1: Describe in detail the selection of the persons included in the study (*i.e.,* study population selection) including filtering based on data quality, data availability and linkage. The selection of included persons can be described in the text and/or by means of the study flow diagram. | Methods, Results and Supplementary Materials (eFigure 1) |
| Descriptive data | 14 | (a) Give characteristics of study participants (*e.g.*, demographic, clinical, social) and information on exposures and potential confounders  (b) Indicate the number of participants with missing data for each variable of interest  (c) *Cohort study* - summarise follow-up time (*e.g.*, average and total amount) | a) Methods, Results and Table 1  b) Table 1 and Supplementary Materials (eFigure 1)  c) Table 1 |  |  |
| Outcome data | 15 | *Cohort study* - Report numbers of outcome events or summary measures over time  *Case-control study* - Report numbers in each exposure category, or summary measures of exposure  *Cross-sectional study* - Report numbers of outcome events or summary measures | Results, Table 1 and Supplementary Materials (eFigure 1) |  |  |
| Main results | 16 | (a) Give unadjusted estimates and, if applicable, confounder-adjusted estimates and their precision (e.g., 95% confidence interval). Make clear which confounders were adjusted for and why they were included  (b) Report category boundaries when continuous variables were categorized  (c) If relevant, consider translating estimates of relative risk into absolute risk for a meaningful time period | a) Results and Supplementary Materials  NA  NA |  |  |
| Other analyses | 17 | Report other analyses done—e.g., analyses of subgroups and interactions, and sensitivity analyses | Results and Supplementary Materials |  |  |
| **Discussion** | | | | | |
| Key results | 18 | Summarise key results with reference to study objectives | Discussion |  |  |
| Limitations | 19 | Discuss limitations of the study, taking into account sources of potential bias or imprecision. Discuss both direction and magnitude of any potential bias | Discussion | RECORD 19.1: Discuss the implications of using data that were not created or collected to answer the specific research question(s). Include discussion of misclassification bias, unmeasured confounding, missing data, and changing eligibility over time, as they pertain to the study being reported. | Discussion |
| Interpretation | 20 | Give a cautious overall interpretation of results considering objectives, limitations, multiplicity of analyses, results from similar studies, and other relevant evidence | Discussion |  |  |
| Generalisability | 21 | Discuss the generalisability (external validity) of the study results | Discussion |  |  |
| **Other Information** | | | | | |
| Funding | 22 | Give the source of funding and the role of the funders for the present study and, if applicable, for the original study on which the present article is based | Acknowledgements |  |  |
| Accessibility of protocol, raw data, and programming code |  | .. |  | RECORD 22.1: Authors should provide information on how to access any supplemental information such as the study protocol, raw data, or programming code. | References to Supplementary Materials throughout |

*Checklist is protected under Creative Commons Attribution ([CC BY](http://creativecommons.org/licenses/by/4.0/)) license.

**eTable 2.** Structural Magnetic Resonance Imaging scanning parameters for each contributing site/cohort within the ENIGMA CHR dataset.

| **Site** | **Bern** | **Toronto** | **Central South** | **Columbia1** | **Columbia3** | **Glasgow** | **Copenhagen** | **Heidelberg1** | **Heidelberg2** |
| --- | --- | --- | --- | --- | --- | --- | --- | --- | --- |
| **Scanner manufacturer** | Siemens | GE | Siemens | GE | GE | Siemens | Philips | Siemens | Siemens |
| **Scanner model** | Magnetom Verio | Discovery MR750 | Skyra | Discovery MR750 | Signa | Trio | Achieva | PET/MR | Tim Trio |
| **Tesla strength** | 3 | 3 | 3 | 3 | 3 | 3 | 3 | 3 | 3 |
| **Acquisition time (seconds)** | - | 1318 | - | 281 | 300 | - | 602 | - | - |
| **TI_ms** | - | 650 | - | 450 | 500 | 900 | - | 900 | 900 |
| **TR_ms** | 7920 | 6736 | 2530 | 7840 | - | 2250 | 10.01 | 2300 | 2300 |
| **TE_ms** | 2.48 | 2.99 | 2.33 | 3.1 | - | 2.6 | 4.6 | 2.98 | 2.98 |
| **Flip angle degree** | 16 | 8 | 7 | 12 | 11 | 9 | 8 | 9 | 9 |
| **Voxel size (mm x mm x mm)** | 1.0 x 1.0 x 1.0 | 0.9 x 0.9 x 0.9 | 1.0 x 1.0 x 1.0 | 0.8 x 0.8 x 0.8 | 0.98 x 0.98 x 1.0 | 1.0 x 1.0 x 1.0 | 0.75 x 0.75 x 0.80 | 1.0 x 1.0 x 1.0 | 1.0 x 1.0 x 1.0 |
| **Freesurfer version** | 6.0.0 | 6.0.0 | 6.0.0 | 6.0.0 | 5.3 | 6.0.0 | 6.0.0 | 6.0.0 | 6.0.0 |
| **Site** | **IDIBAPS1** | **IDIBAPS2** | **IDIBAPS3** | **London1** | **London2** | **Maastricht1** | **Maastricht2** | **Melbourne1** | **Melbourne2** |
| **Scanner manufacturer** | Siemens | Siemens | Siemens | GE | GE | Philips | Philips | Siemens | Siemens |
| **Scanner model** | Tim Trio | Prisma Fit | Prisma Fit | Discovery MR750 | Signa HDx | Intera | Achieva | Trio | Trio |
| **Tesla strength** | 3 | 3 | 3 | 3 | 3 | 3 | 3 | 3 | 3 |
| **Acquisition time (seconds)** | - | - | - | - | - | 1176 | 1176 | - | 548.4 |
| **TI_ms** | 900 | 900 | 900 | 400 | - | 1000 | 1000 | - | 900 |
| **TR_ms** | 2300 | 2300 | 2300 | 7.31 | 7.144 | 2250 | 2250 | 1900 | 2300 |
| **TE_ms** | 3.01 | 3.01 | 2.98 | 3.02 | 2.85 | 4.6 | 4.6 | 2.15 | 2.98 |
| **Flip angle degree** | 9 | 9 | 9 | 11 | 20 | 8 | 8 | 90 | 9 |
| **Voxel size (mm x mm x mm)** | 0.94 x 0.94 x 1.0 | 0.94 x 0.94 x 1.0 | 0.94 x 0.94 x 1.2 | 1.1 x 1.1 x 1.2 | 1.1 x 1.1 x 1.1 | 1.17 x 1.17 x 1.20 | 1.2 x 0.8 x 0.8 | 0.5 x 0.5 x 1.0 | 1.0 x 1.0 x 1.2 |
| **Freesurfer version** | 6.0.0 | 6.0.0 | 6.0.0 | 6.0.0 | 6.0.0 | 6.0.0 | 6.0.0 | 6.0.0 | 6.0.0 |

| **Site** | **Melbourne3** | **Melbourne4** | **Mexico City** | **MHRC** | **MPRC1** | **MPRC2** | **Mt Sinai** | **Newcastle** | **Oslo1** |
| --- | --- | --- | --- | --- | --- | --- | --- | --- | --- |
| **Scanner manufacturer** | GE | GE | GE | Philips | Siemens | Siemens | Siemens | Siemens | GE |
| **Scanner model** | Signa | LX Horizon | Signa HDx | Achieva | Trio | Prisma | Skyra | Avanto | Signa HDxt |
| **Tesla strength** | 1.5 | 3 | 3 | 3 | 3 | 3 | 3 | 1.5 | 3 |
| **Acquisition time (seconds)** | - | - | 1175 | 443 | 720 | 396 | 422 | 346.5 | 428 |
| **TI_ms** | - | - | 450 | - | 1000 | 1000 | 1000 | 1100 | 450 |
| **TR_ms** | 14.3 | 36 | 1340 | 8.2 | 2400 | 2400 | 2400 | 1980 | 7800 |
| **TE_ms** | 3.3 | 9 | 5.7 | 3.7 | 2.2 | 2.2 | 2.2 | 4.3 | 2.956 |
| **Flip angle degree** | 30 | 30 | 20 | 8 | 8 | 8 | 8 | 12 | 12 |
| **Voxel size (mm x mm x mm)** | 0.94 x 0.94 x 1.5 | 0.49 x 0.49 x 2 | 1.2 x 1.2 x 1.2 | 0.83 x 0.83 x 1.0 | 0.8 x 0.8 x 0.8 | 0.8 x 0.8 x 0.8 | 0.8 x 0.8 x 0.8 | 0.98 x 0.98 x 1 | 1.0 x 1.0 x 1.2 |
| **Freesurfer version** | 6.0.0 | 6.0.0 | 6.0.0 | 6.0.0 | 6.0.0 | 6.0.0 | 6.0.0 | 6.0.0 | 5.3 |
| **Site** | **Oslo2** | **Pitt** | **Rush** | **SNUH** | **Singapore** | **Stavanger** | **Toho** | **UCSF** | **Tokyo1** |
| **Scanner manufacturer** | GE | Siemens | Siemens | Siemens | Siemens | GE | Toshiba | Siemens | GE |
| **Scanner model** | Discovery MR750 | Prisma | Verio | Megnetom TrioTim | Tim Trio | 450 Discovery | EXCELART Vantage | Magnetom TrioTim | Signa HDx |
| **Tesla strength** | 3 | 3 | 3 | 3 | 3 | 1.5 | 1.5 | 3 | 3 |
| **Acquisition time (seconds)** | 283 | 720 | 363 | 234 | 303 | 362 | - | 554 | - |
| **TI_ms** | 450 | 1000 | 1100 | - | 900 | 450 | - | 900 | - |
| **TR_ms** | 8.16 | 2400 | 2530 | 1670 | 2300 | 7.9 | - | 2300 | 6.8 |
| **TE_ms** | 3.18 | 2.2 | 2.27 | 1.89 | 3 | 3.1 | - | 2.95 | 1.94 |
| **Flip angle degree** | 12 | 8 | 7 | 9 | 9 | 12 | 35 | 9 | 20 |
| **Voxel size (mm x mm x mm)** | 1.0 x 1.0 x 1.0 | 0.8 x 0.8 x 0.8 | 1.0 x 1.0 x 1.0 | 1.0 x 0.98 x 0.98 | 1.0 x 1.0 x 1.0 | - | 0.98 x 0.98 x 1.0 | 1.0 x 1.0 x 1.2 | 1.0 x 1.0 x 1.0 |
| **Freesurfer version** | 5.3 | 6.0.0 | 6.0.0 | 6.0.0 | 6.0.0 | 5.3 | 5.2 | 5.1 | 6.0.0 |

| **Site** | **Tokyo2** | **Toyoma1** | **Toyoma2** | **Amsterdam** | **Zurich** |
| --- | --- | --- | --- | --- | --- |
| **Scanner manufacturer** | GE | Siemens | Siemens | Philips | Philips |
| **Scanner model** | Discovery MR750W | Magnetom Vision | Magnetom Verio | Image MR Series | Philips Achieva TX |
| **Tesla strength** | 3 | 1.5 | 3 | 3 | 3 |
| **Acquisition time (seconds)** | - | - | - | - | 449 |
| **TI_ms** | - | - | - | - | 1000 |
| **TR_ms** | 8.46 | 2400 | 2300 | 8280 | 8.3 |
| **TE_ms** | 3.25 | 5 | 2.9 | 3.8 | 3.8 |
| **Flip angle degree** | 20 | 40 | 9 | - | 8 |
| **Voxel size (mm x mm x mm)** | 1.0 x 1.0 x 1.0 | 1.0 x 1.0 x 1.0 | 1.0 x 1.0 x 1.2 | - | 1.0 x 1.0 x 1.0 |
| **Freesurfer version** | 6.0.0 | 6.0.0 | 6.0.0 | 6.0.0 | 6.0.0 |

**eMethods 1.** Leave-one-site-out resampling method.

Furthermore, in light of the variability in recruitment strategies, assessment methods and the scanning protocol adopted at each contributing geographical site, it was also necessary to conduct supplementary analyses to investigate ‘site effects’. We employed the ‘leave-one-site-out’ resampling method in which we repeated the regional VR analyses (CHR vs HC), leaving one site out at a time. We planned to conduct these analyses for all regions of interest which were found to be statistically significant in our original analyses. Regions which fail to demonstrate a statistically significant effect (*p*<.01) across more than 10% of the sites (i.e. 3 sites), were deemed to be less robust and untrustworthy, in line with previous research.^2^

**eMethods 2.** Age-, sex- and site-matched analyses.

Due to the presence of minor differences in age and sex between the CHR-P and HC sample, alongside much variation in geographical site, we also planned to repeat the key VR analyses in a matched sample to determine whether significant findings remained robust when the sample was matched for these potentially confounding variables. We planned to use the MatchIt() function in R to match the two cohorts across age, sex, and site, utilising the ‘exact’ matching argument^3,4^ before repeating the key VR analyses, comparing the reduced CHR-P sample with the matched HC sample.

**eMethods 3.** Z-scores indexing the baseline summed severity of symptoms on the CAARMS and SIPS assessments.

The Comprehensive Assessment of At-Risk Mental States (CAARMS)^5^ assessment and the Structured Interview for Prodromal Symptoms (SIPS)^6^ assessment represent different assessment and scoring procedures to reach classification of an at-risk mental state. Therefore, in order to facilitate the investigation of potential effects of psychopathology on PBSI scores across individuals at CHR-P, we converted the CAARMS summed psychosis severity score and SIPS summed psychosis severity score into z-scores, respectively.

**eFigure 1.** Flow diagram to represent the included/excluded participants and the final sample, according to a) individuals at CHR-P, and b) control participants.

1. ***b)***

CHR-P participants in ENIGMA dataset (n=1792)

CHR-P Participants with >5% missing MRI data (n=139)

Final CHR-P sample for analysis (n=1579)

Transition to psychosis (n=226)

No transition to psychosis (n=1095)

Unknown outcome

(n=258)

CHR-P participants excluded due to IRB^a^ restrictions (n=74)

Control participants in ENIGMA dataset (n=1377)

Control participants with >5% missing MRI data (n=75)

Final control sample for analysis (n=1243)

Control participants excluded due to IRB^a^ restrictions (n=59)

***^a^*** *IRB = Institutional Review Board: two sites within the ENIGMA CHR-P working group were excluded from this current analysis due to local IRB restrictions (Sant Joan Déu Barcelona Hospital and Columbia3), leading to a smaller sample size within these current analyses compared with the original ENIGMA CHR-P working group analyses.*^2^

**eTable 3.** Sample characteristics for the CHR-P-Transition (CHR-T) and CHR-P-No Transition (CHR-NT) groups.

|  | **CHR-T (N=226)** | **CHR-NT (N=1097)** |
| --- | --- | --- |
| Age in years, *mean (SD)* | 19.93 (4.35) | 20.69 (4.65) |
| Sex, *M/F* | 131/95 | 563/534 |
| Follow-up durations in months, *mean (SD)* | 19.21 (17.79) | 31.30 (34.90) |
| Typical antipsychotics, *n (%)* | 2 (0.88%) | 11 (1.00%) |
| Atypical antipsychotics, *n (%)* | 29 (12.83%) | 135 (12.31%) |

**eFigure 2:** Forest plot of the Coefficient of Variation (CV) ratio of Surface Area (SA) in individuals at CHR-P compared with HC.


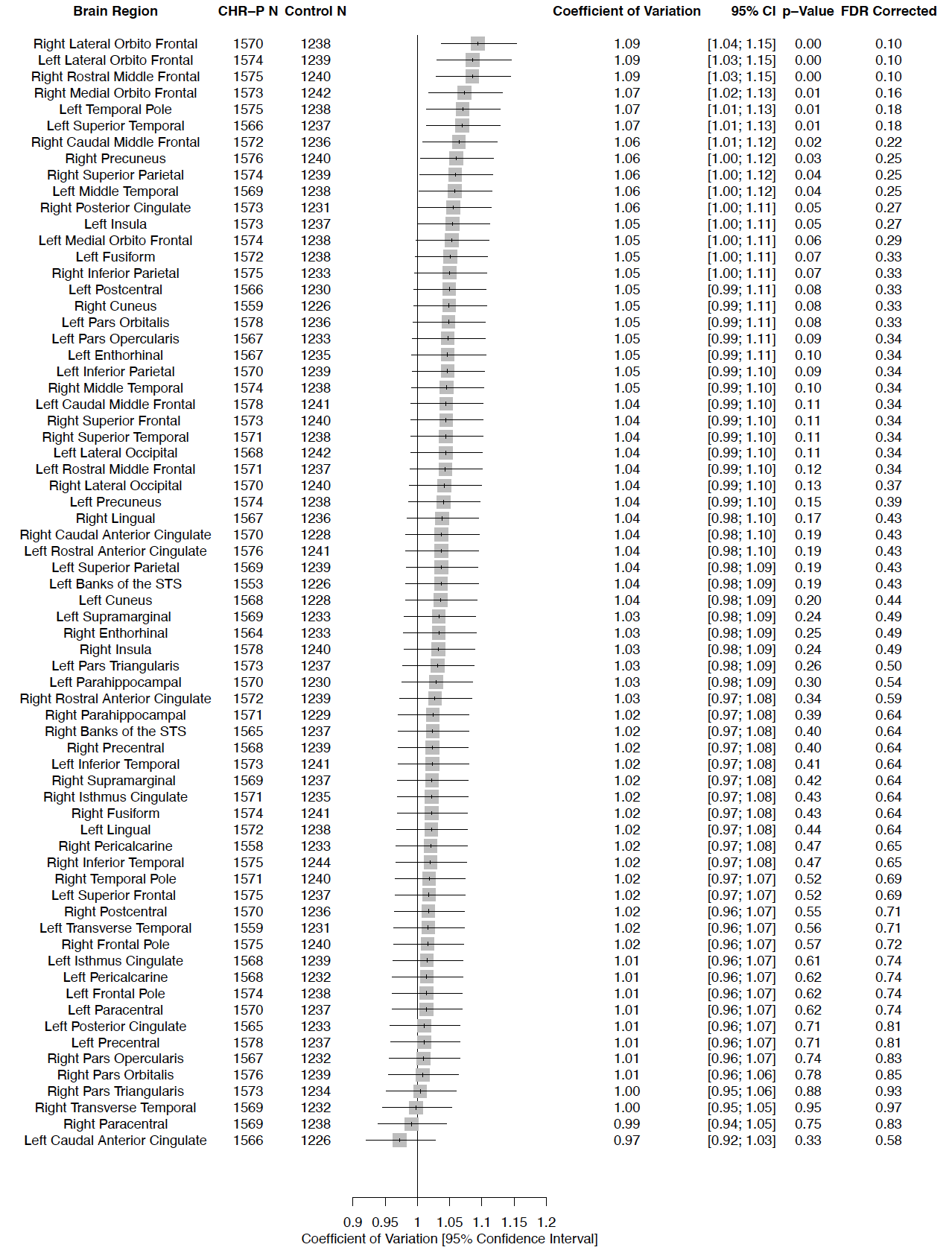


**eFigure 3:** Forest plot of the Coefficient of Variation (CV) ratio of Cortical Thickness (CT) in individuals at CHR-P compared with HC.


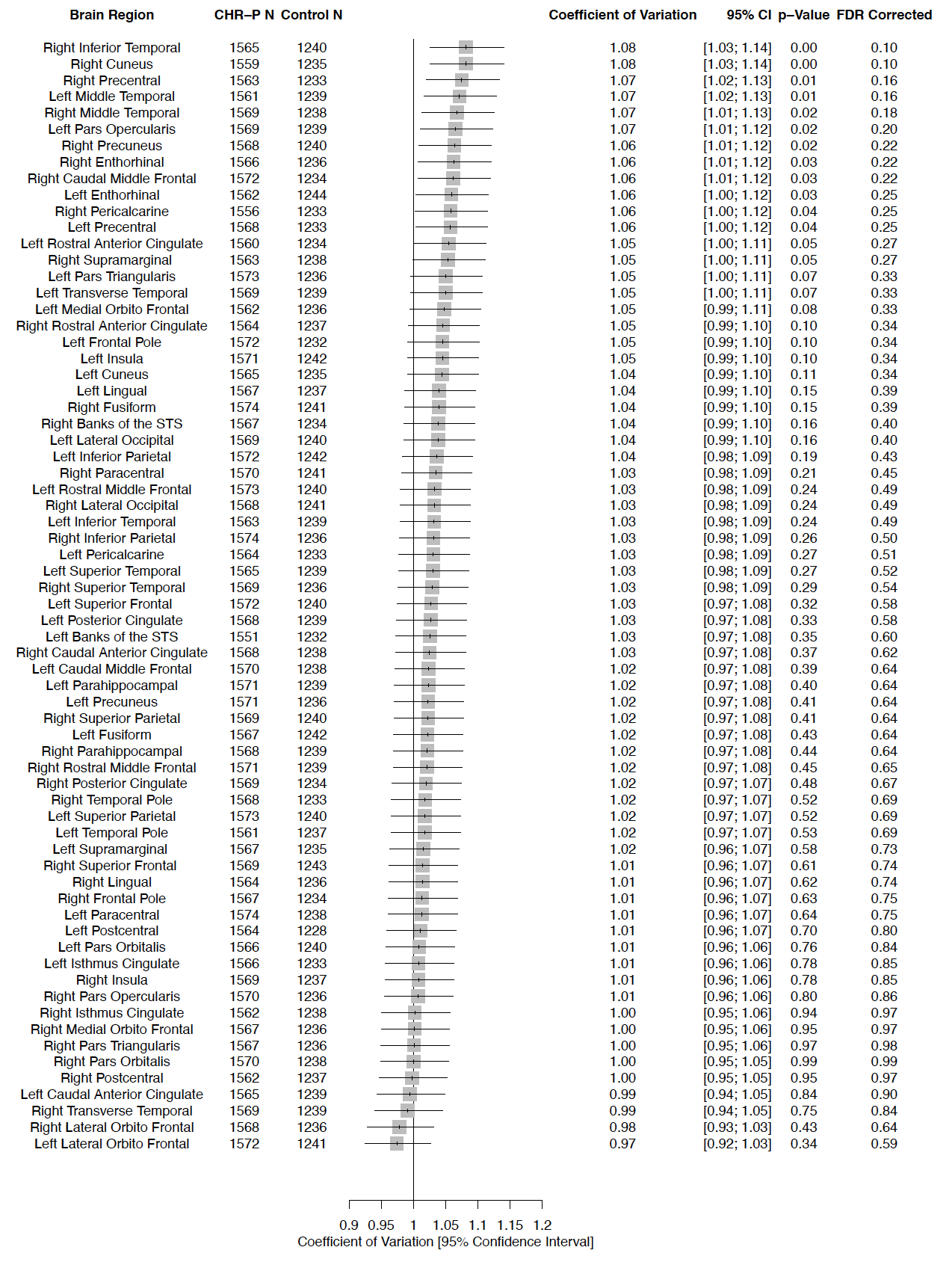


**eFigure 4:** Forest plot of the Coefficient of Variation (CV) ratio of Subcortical Volume (SV) in individuals at CHR-P compared with HC.


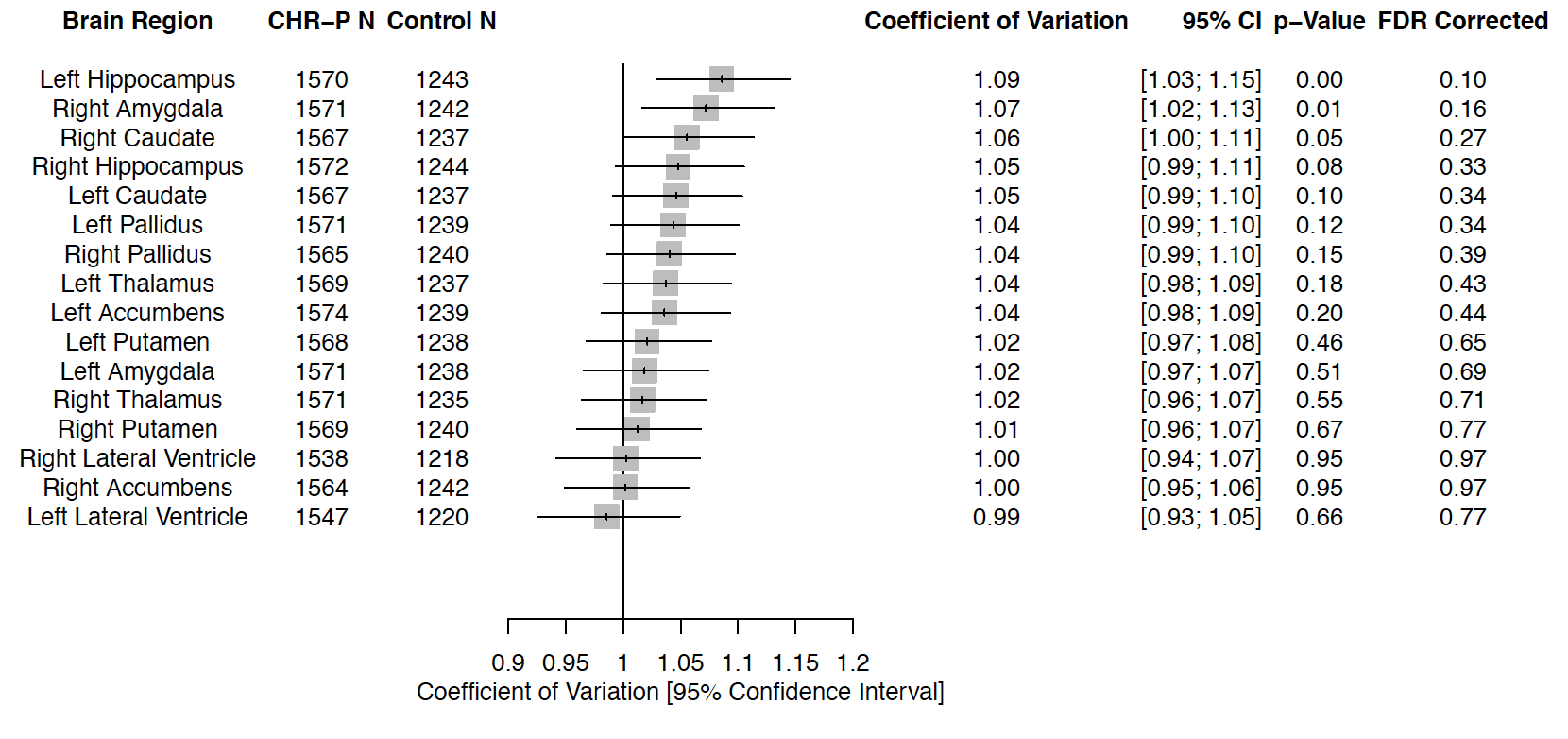


**eFigure 5:** Forest plot of the Variability Ratio (VR) of Intra-Cranial Volume (ICV) in individuals at CHR-P compared with HC.


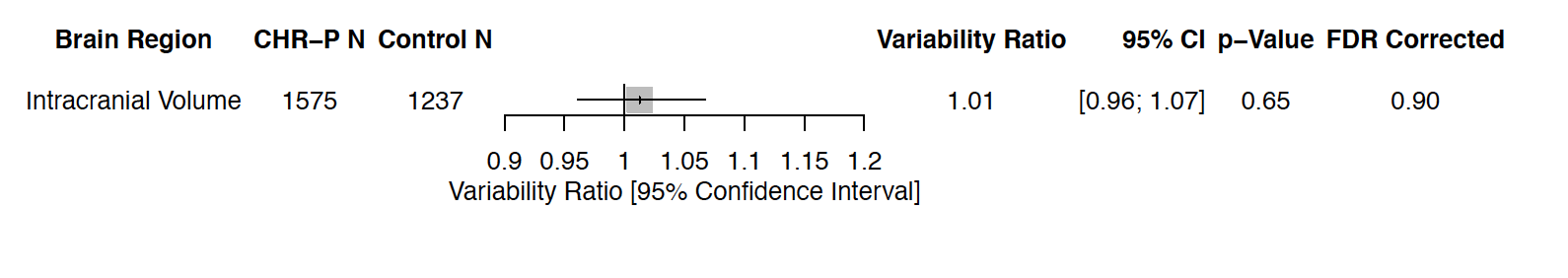


**eFigure 6:** Forest plot of the Coefficient of Variation (CV) ratio of Intra-Cranial Volume (ICV) in individuals at CHR-P compared with HC.


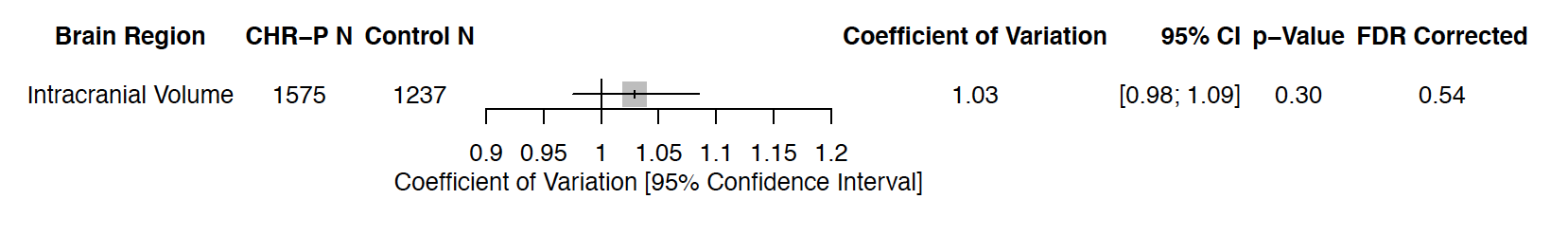


**eFigure 7:** Forest plot of the Variability Ratio (VR) of cortical Surface Area (SA) in individuals at CHR-P who subsequently transition to psychosis (CHR-T) compared with those who did not (CHR-NT).


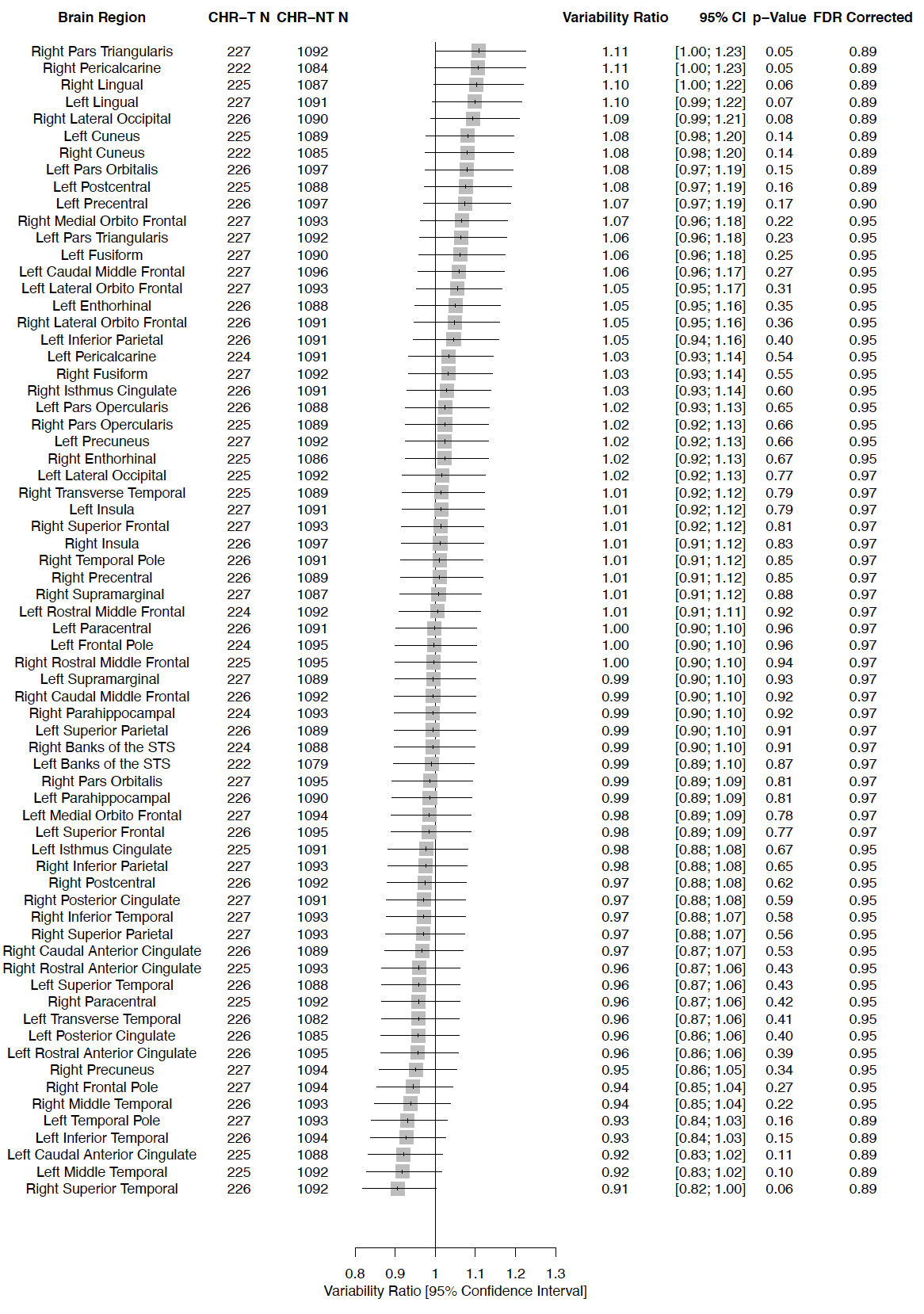


**eFigure 8:** Forest plot of the Coefficient of Variation (CV) ratio of cortical Surface Area (SA) in individuals at CHR-P who subsequently transition to psychosis (CHR-T) compared with those who did not (CHR-NT).


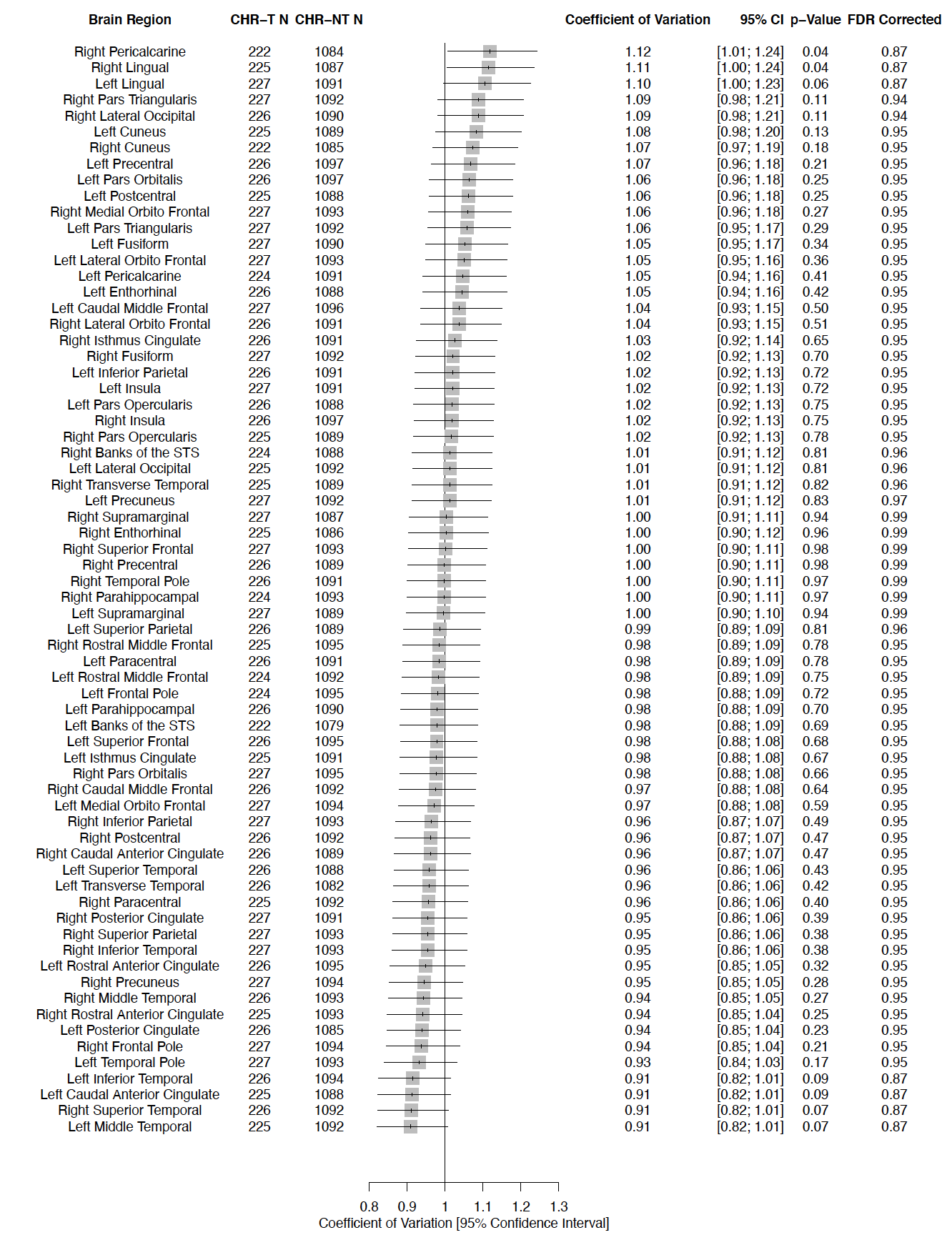


**eFigure 9:** Forest plot of the Variability Ratio (VR) of Cortical Thickness (CT) in individuals at CHR-P who subsequently transition to psychosis (CHR-T) compared with those who did not (CHR-NT).


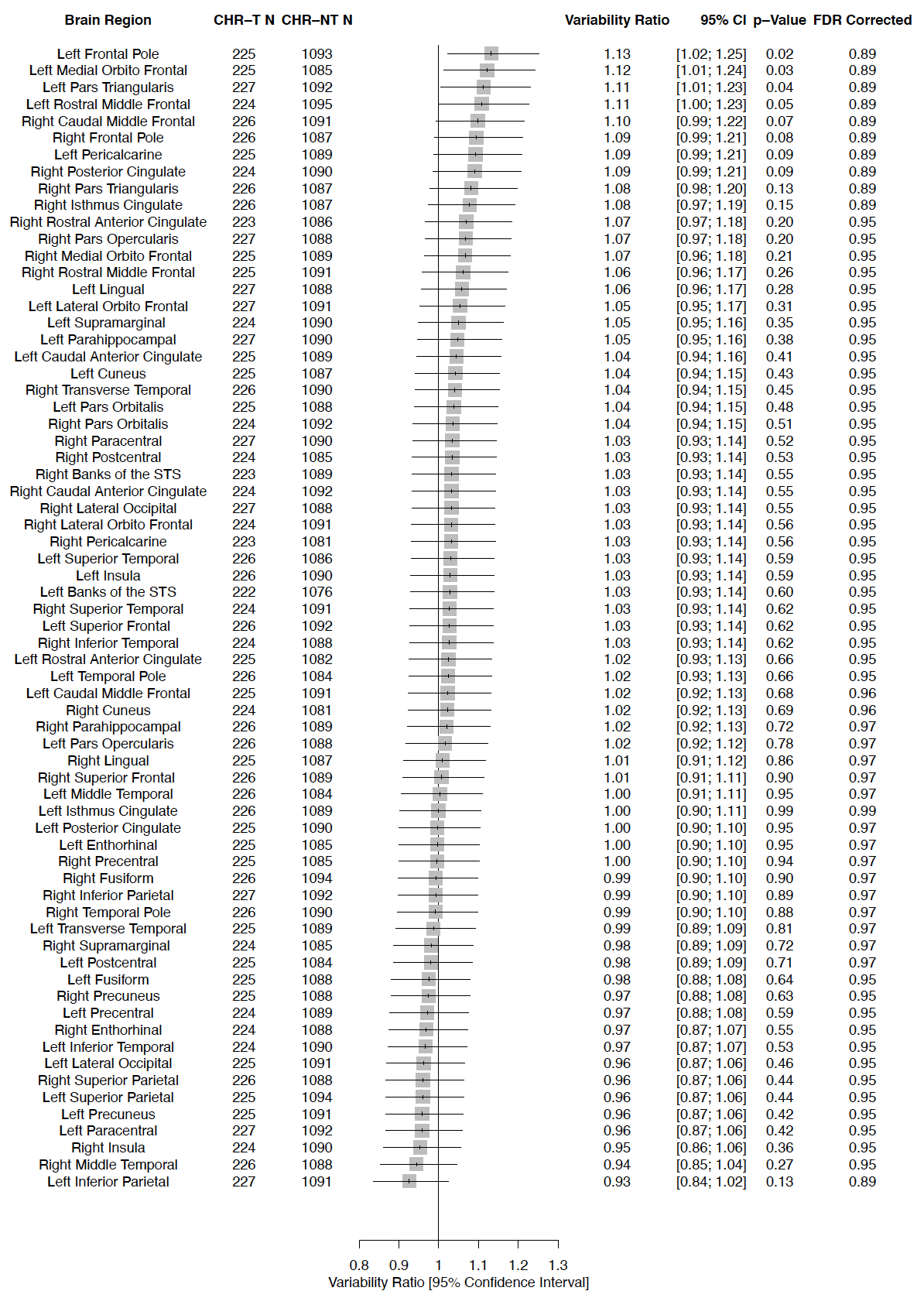


**eFigure 10:** Forest plot of the Coefficient of Variation (CV) ratio of Cortical Thickness (CT) in individuals at CHR-P who subsequently transition to psychosis (CHR-T) compared with those who did not (CHR-NT).


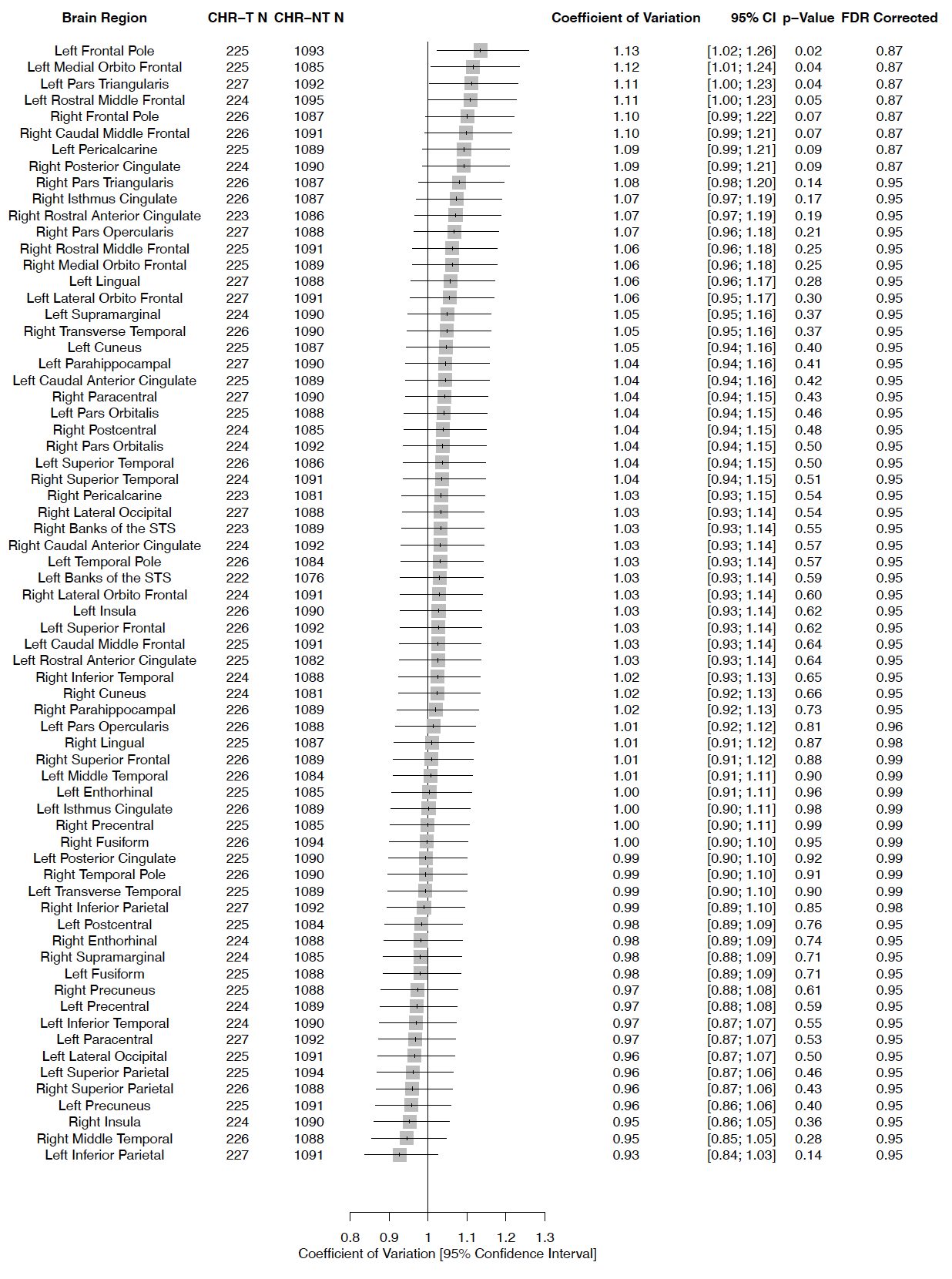


**eFigure11:** Forest plot of the Variability Ratio (VR) of Subcortical Volume (SV) in individuals at CHR-P who subsequently transition to psychosis (CHR-T) compared with those who did not (CHR-NT).


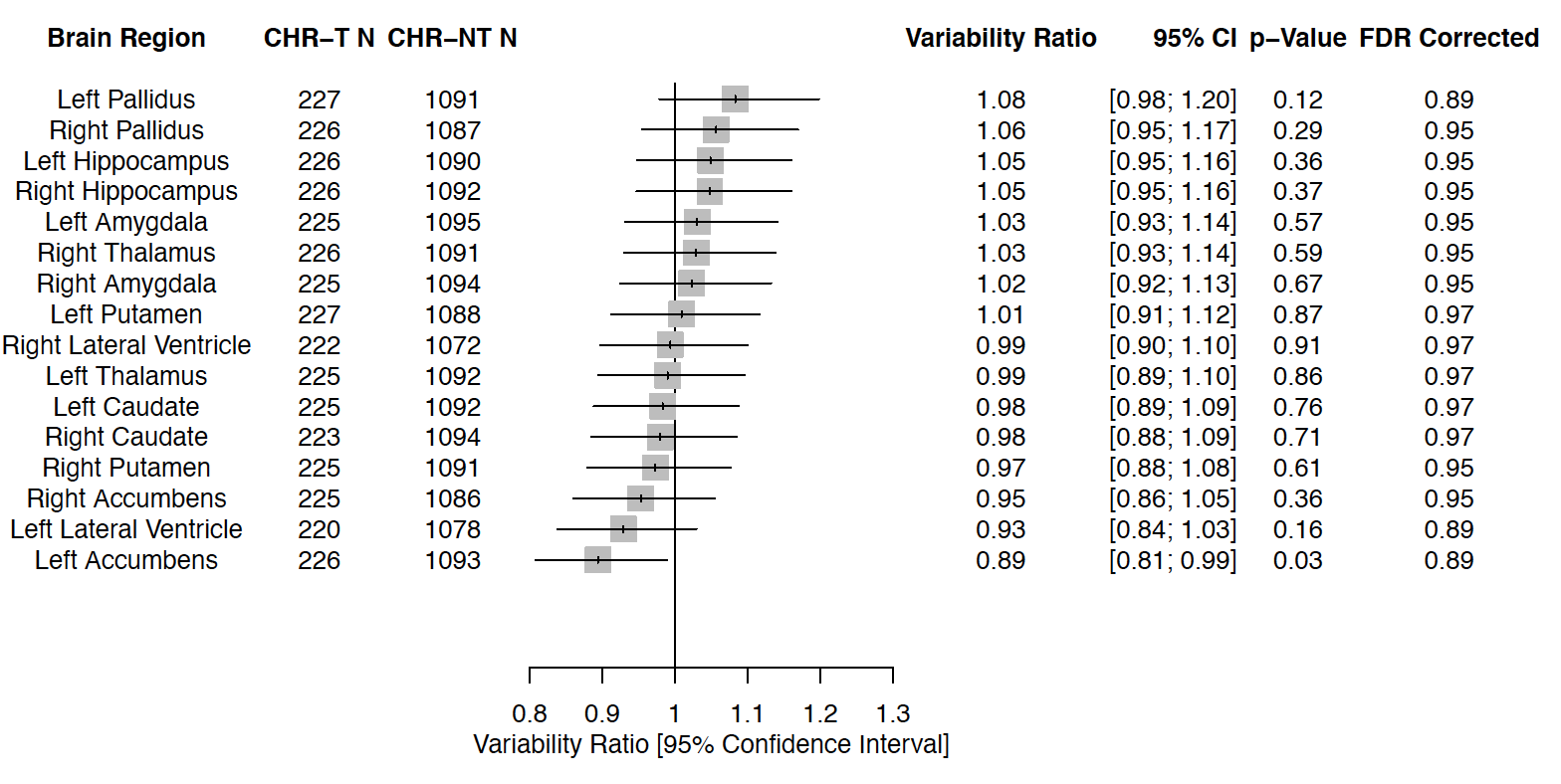


**eFigure12:** Forest plot of the Coefficient of Variation (CV) ratio of Subcortical Volume (SV) in individuals at CHR-P who subsequently transition to psychosis (CHR-T) compared with those who did not (CHR-NT).


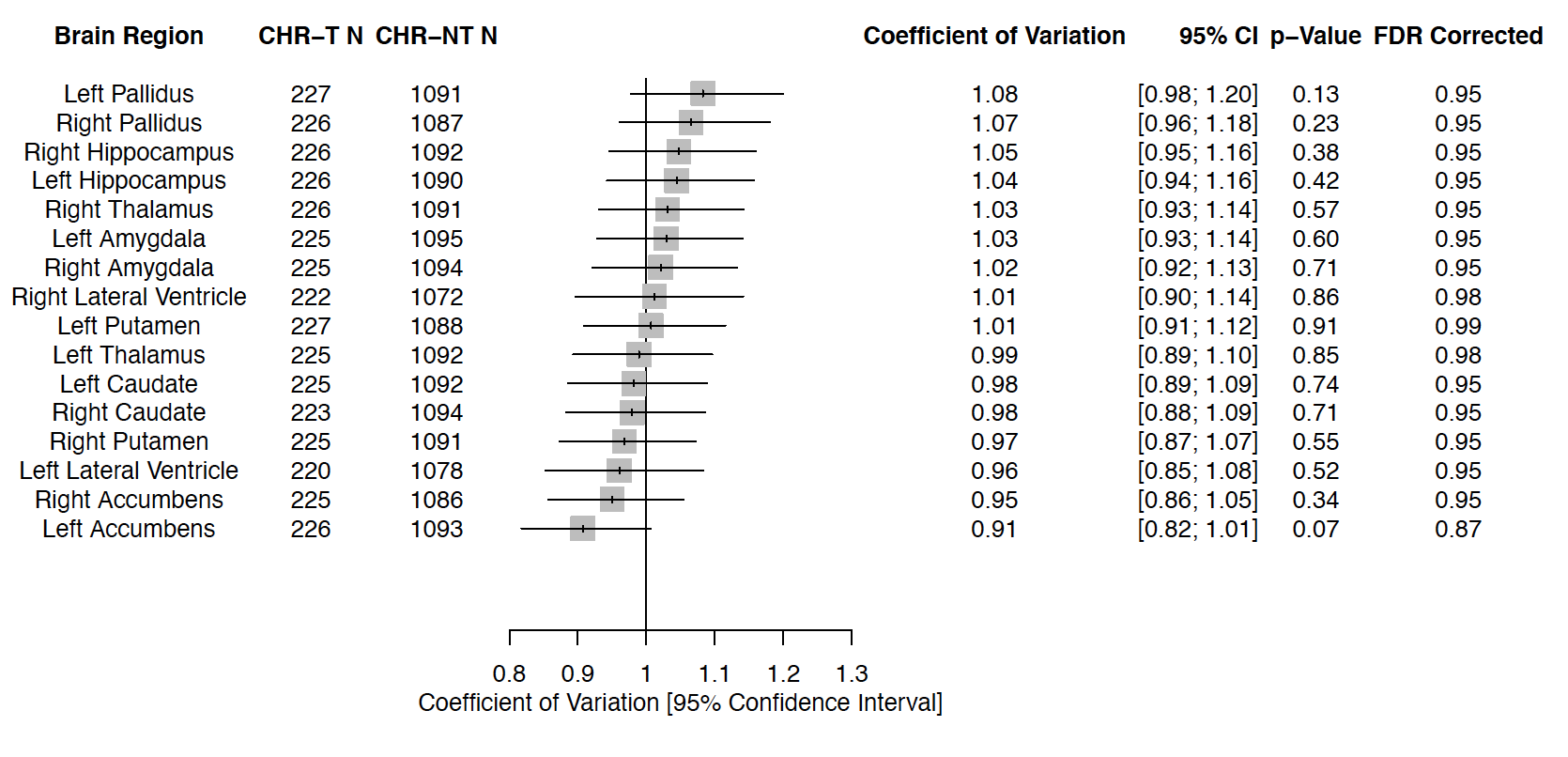


**eFigure13:** Forest plot of the Variability Ratio (VR) of Intra-Cranial Volume (ICV) in individuals at CHR-P who subsequently transition to psychosis (CHR-T) compared with those who did not (CHR-NT).


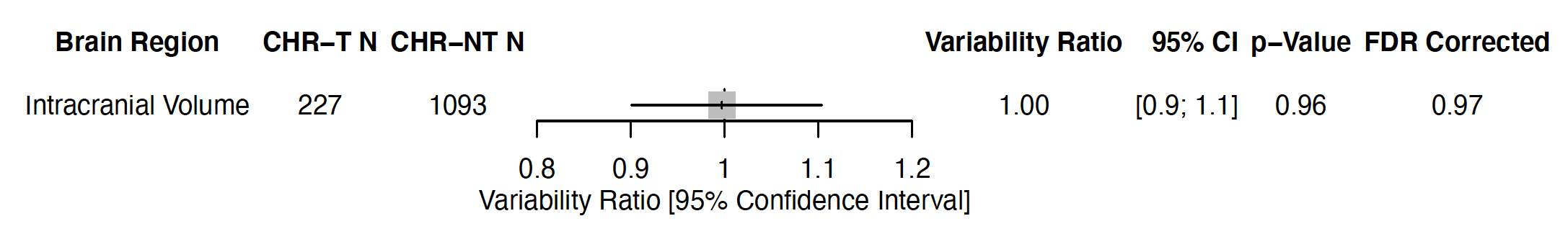


**eFigure14:** Forest plot of the Coefficient of Variation (CV) ratio of Intra-Cranial Volume (ICV) in individuals at CHR-P who subsequently transition to psychosis (CHR-T) compared with those who did not (CHR-NT).


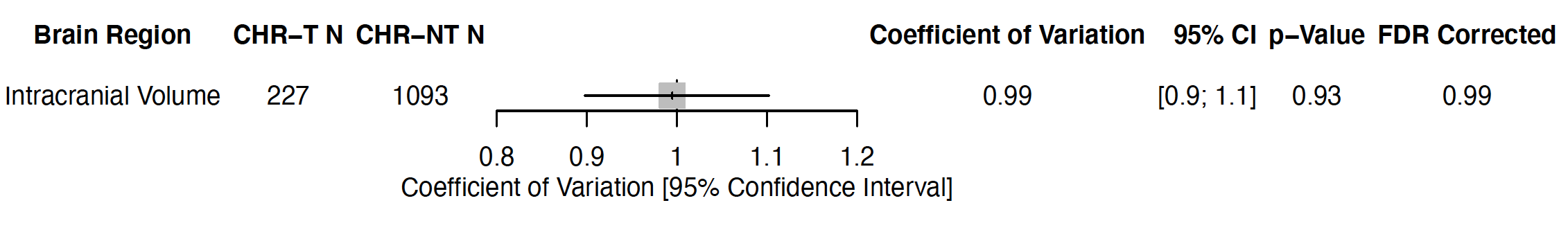


**eFigure 15:** Forest plot of the Variability Ratio (VR) of cortical Surface Area (SA) in APS-allocated individuals compared with HC.


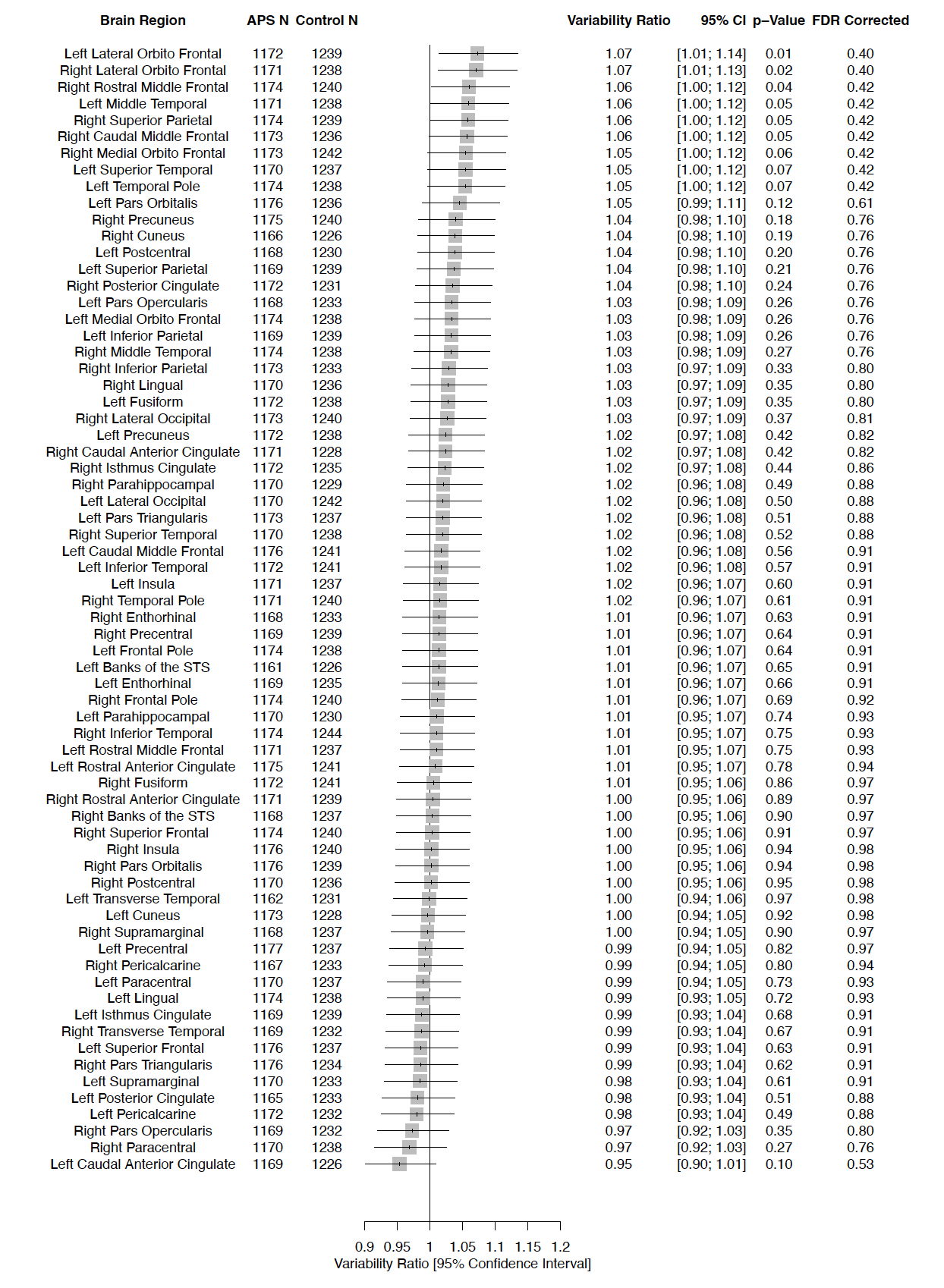


**eFigure 16:** Forest plot of the Coefficient of Variation (CV) ratio of cortical Surface Area (SA) in APS-allocated individuals compared with HC.


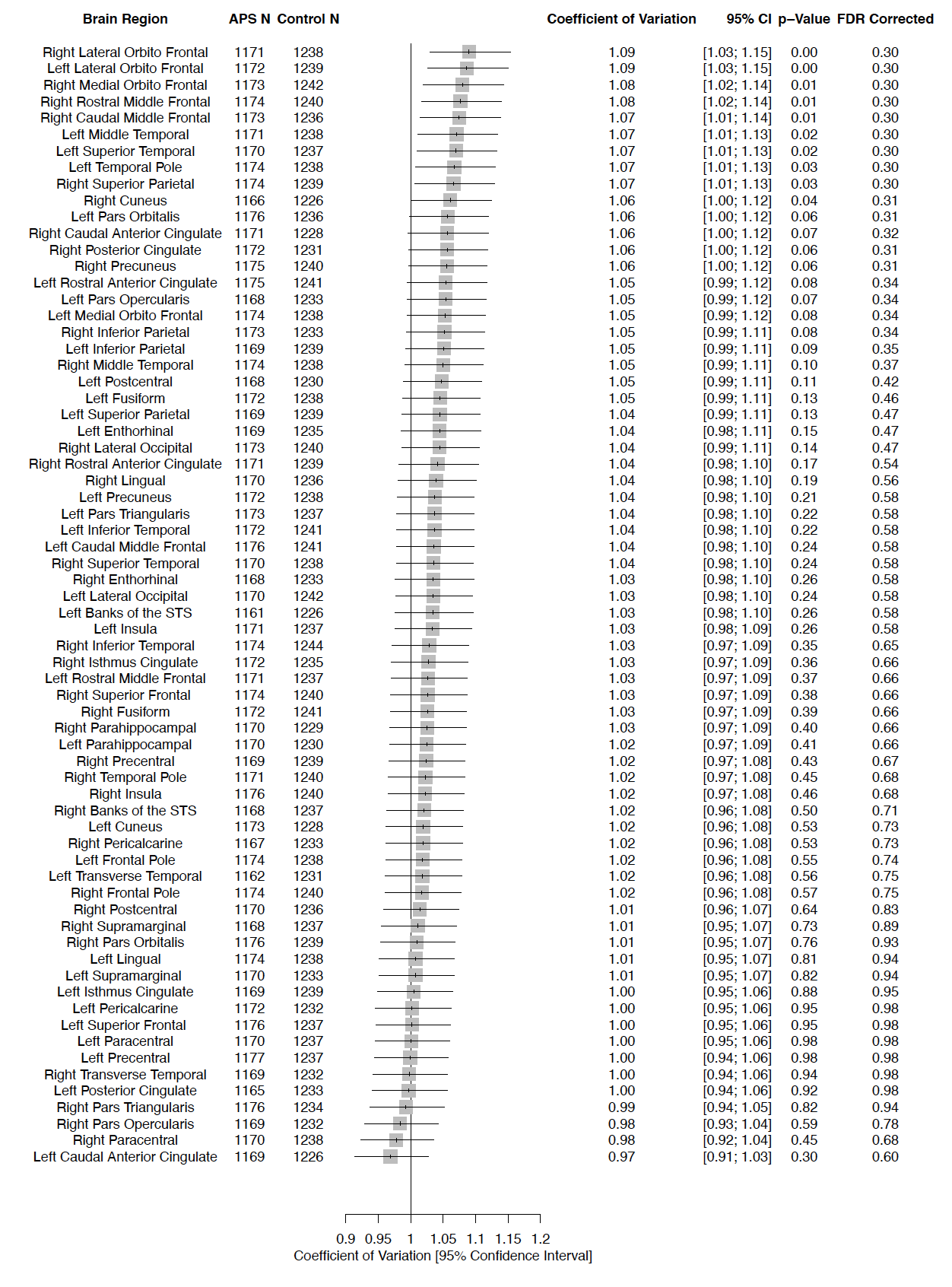


**eFigure 17:** Forest plot of the Variability Ratio (VR) of Cortical Thickness (CT) in APS-allocated individuals compared with HC.


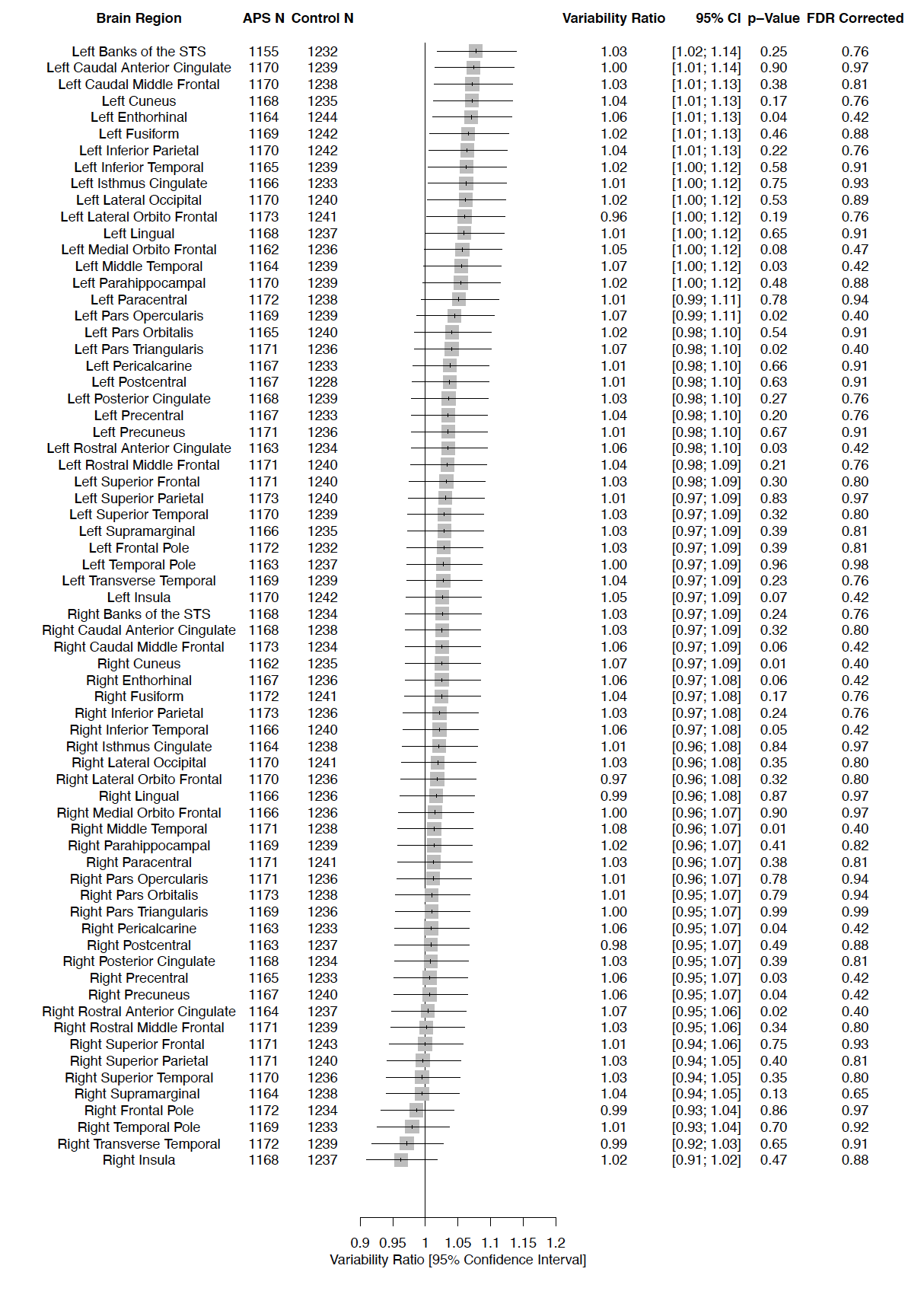


**eFigure 18:** Forest plot of the Coefficient of Variation (CV) ratio of Cortical Thickness (CT) in APS-allocated individuals compared with HC.


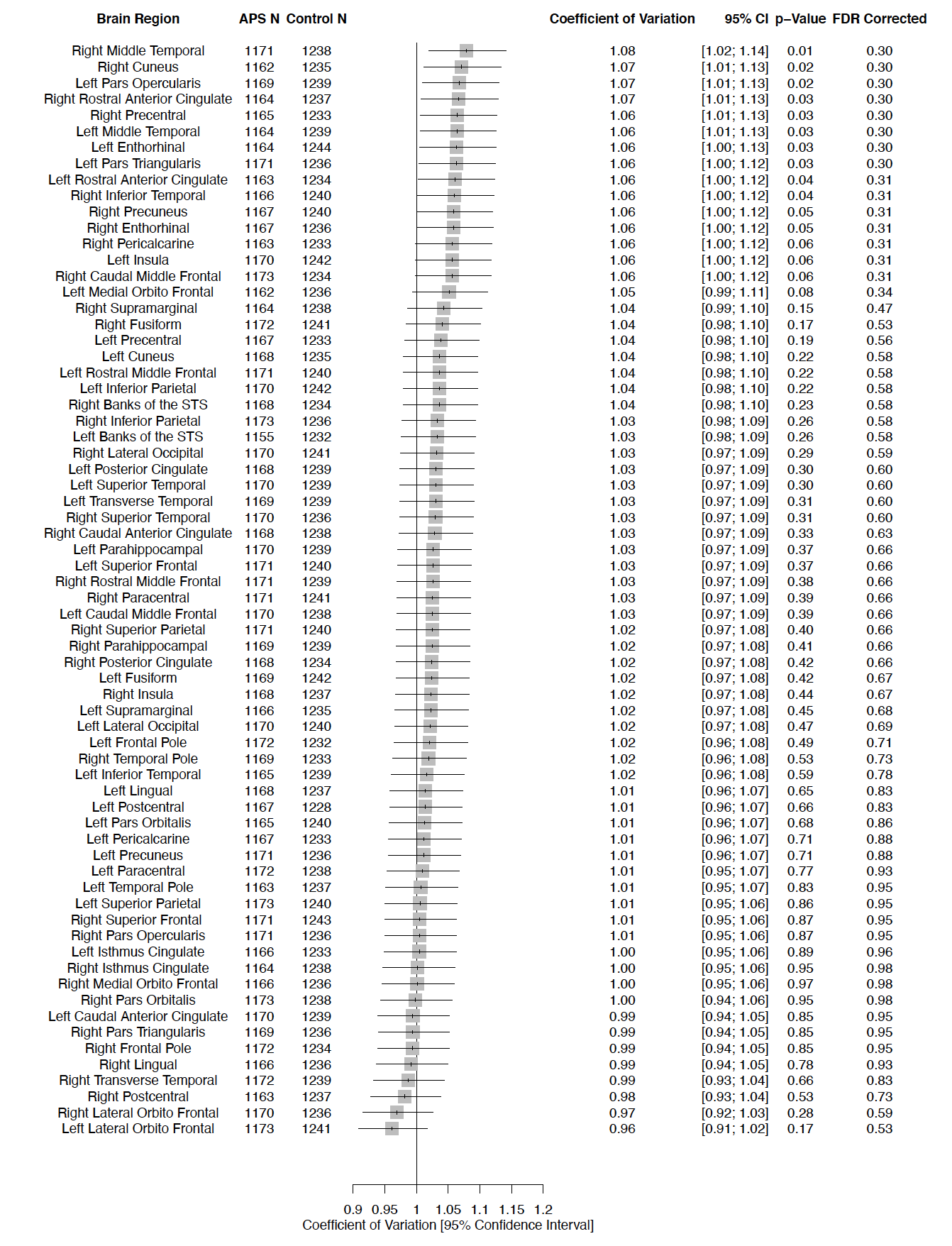


**eFigure 19:** Forest plot of the Variability Ratio (VR) of Subcortical Volume (SV) in APS-allocated individuals compared with HC.


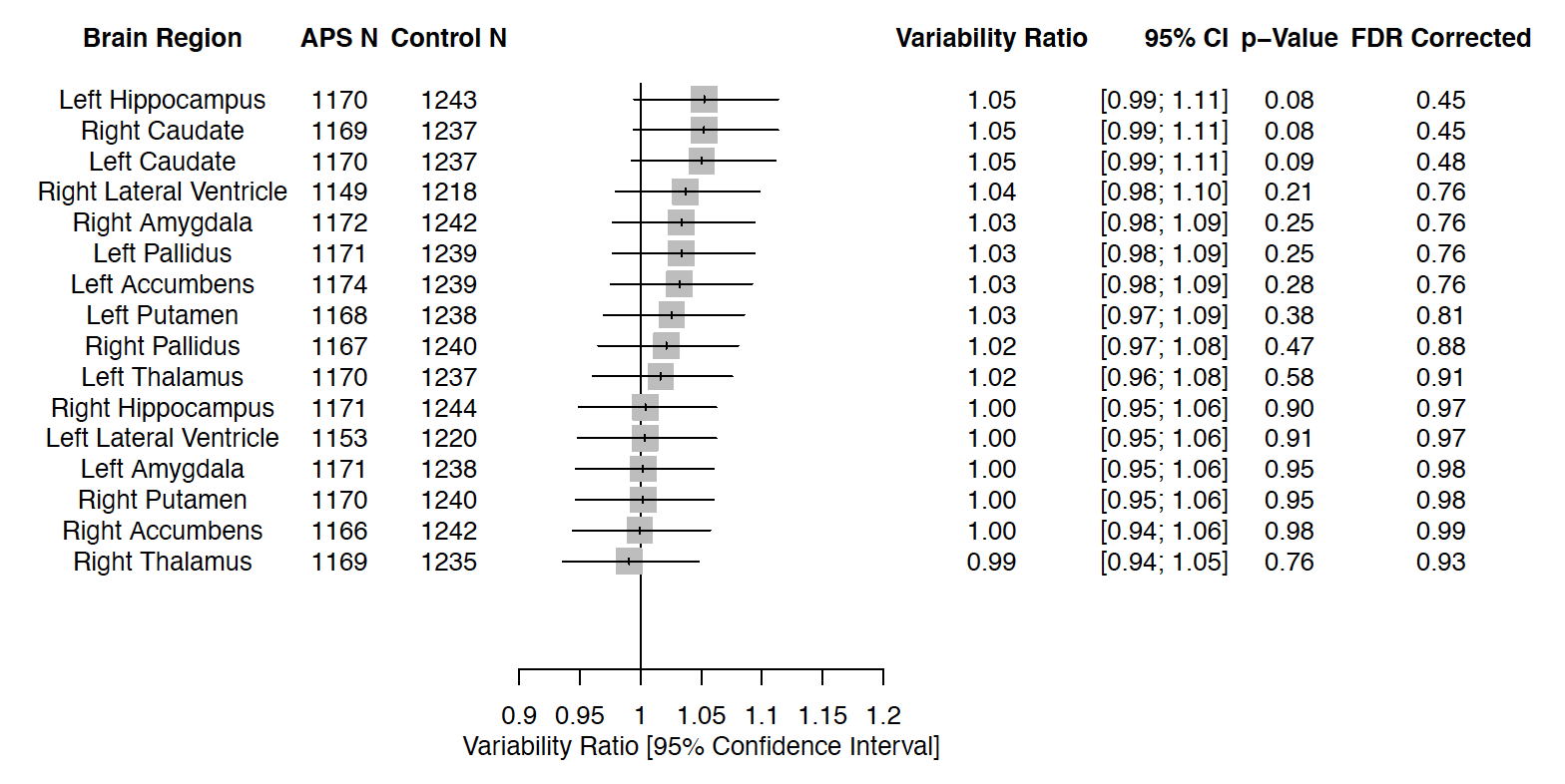


**eFigure 20:** Forest plot of the Coefficient of Variation (CV) ratio of Subcortical Volume (SV) in APS-allocated individuals compared with HC.


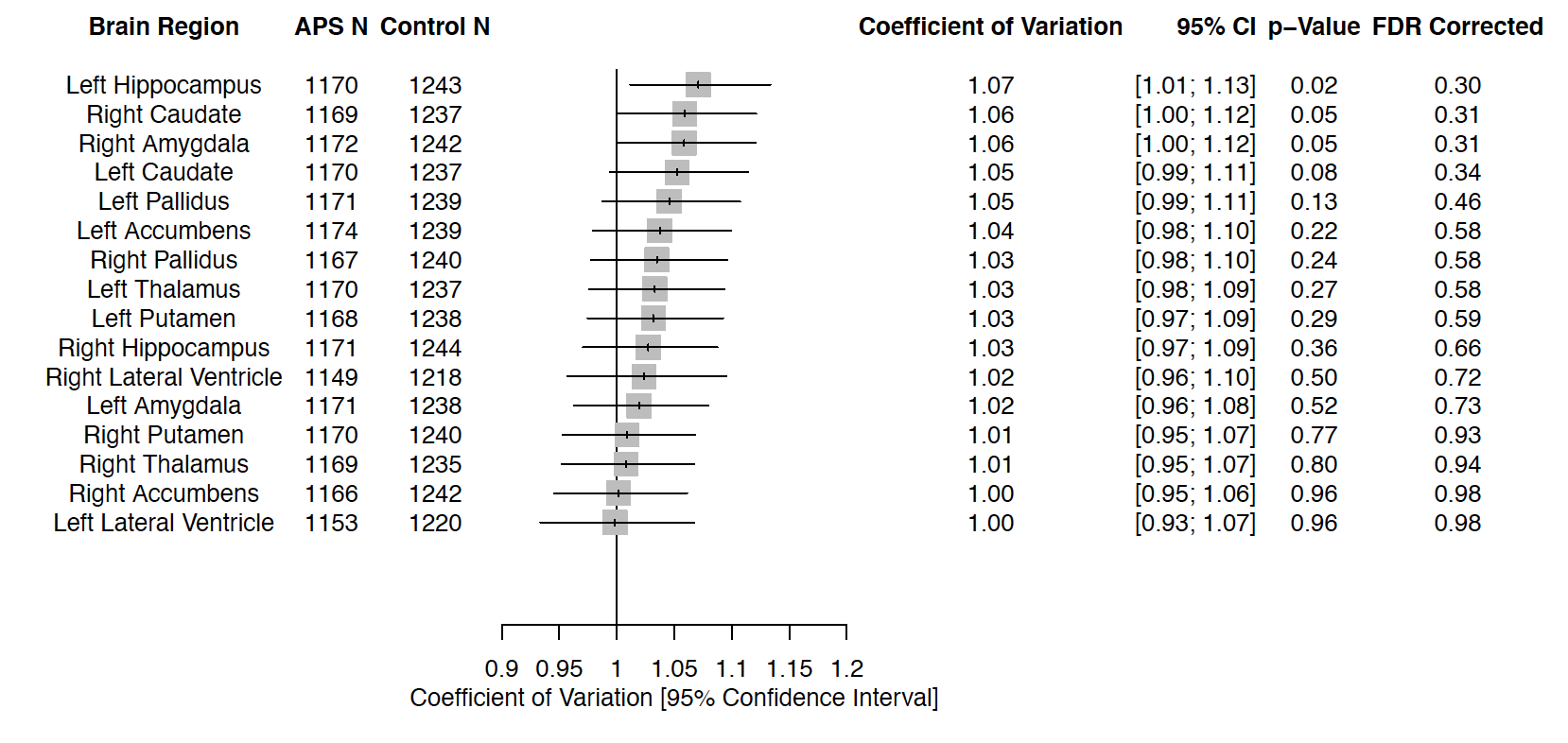


**eFigure 21:** Forest plot of the Variability Ratio (VR) of Intra-Cranial Volume (ICV) in APS-allocated individuals compared with HC.


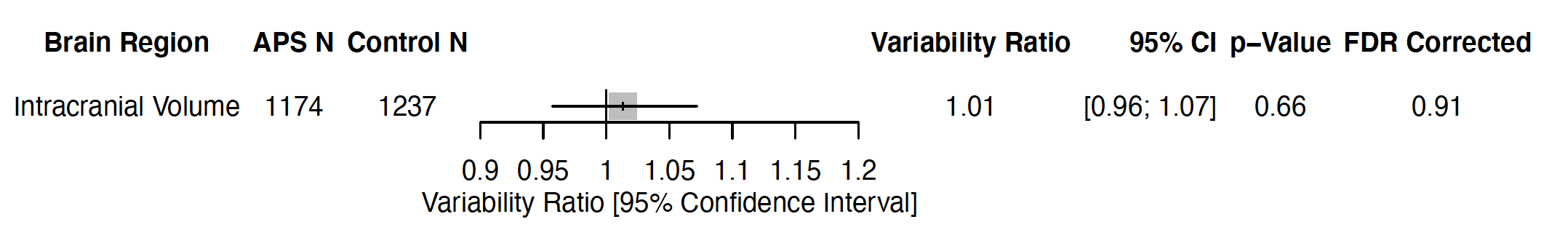


**eFigure 22:** Forest plot of the Coefficient of Variation (CV) ratio of Intra-Cranial Volume (ICV) in APS-allocated individuals compared with HC.


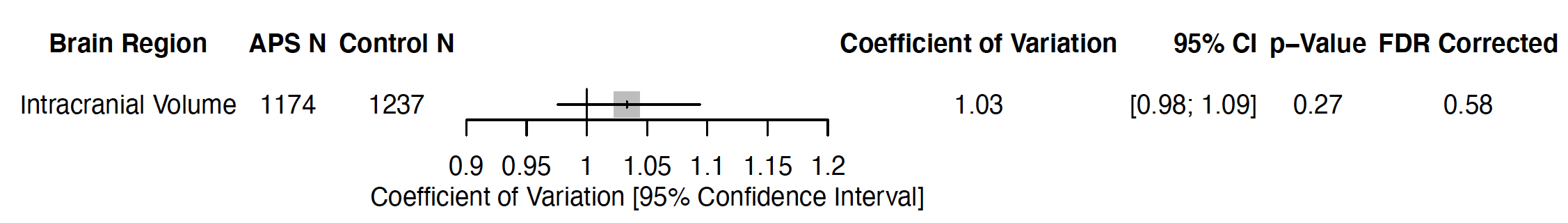


**eReferences**

1. Benchimol EI, Smeeth L, Guttmann A, et al. The REporting of studies Conducted using Observational Routinely-collected health Data (RECORD) Statement. *PLOS Med*. 2015;12(10):e1001885. doi:10.1371/journal.pmed.1001885

2. ENIGMA Clinical High Risk for Psychosis Working Group, Jalbrzikowski M, Hayes RA, et al. Association of Structural Magnetic Resonance Imaging Measures With Psychosis Onset in Individuals at Clinical High Risk for Developing Psychosis: An ENIGMA Working Group Mega-analysis. *JAMA Psychiatry*. 2021;78(7):753. doi:10.1001/jamapsychiatry.2021.0638

3. Ho DE, Imai K, King G, Stuart EA. **MatchIt** : Nonparametric Preprocessing for Parametric Causal Inference. *J Stat Softw*. 2011;42(8). doi:10.18637/jss.v042.i08

4. Stuart EA. Matching Methods for Causal Inference: A Review and a Look Forward. *Stat Sci*. 2010;25(1). doi:10.1214/09-STS313

5. Yung AR, Yung AR, Pan Yuen H, et al. Mapping the Onset of Psychosis: The Comprehensive Assessment of At-Risk Mental States. *Aust N Z J Psychiatry*. 2005;39(11-12):964-971. doi:10.1080/j.1440-1614.2005.01714.x

6. Miller TJ, McGlashan TH, Rosen JL, et al. Prodromal Assessment With the Structured Interview for Prodromal Syndromes and the Scale of Prodromal Symptoms: Predictive Validity, Interrater Reliability, and Training to Reliability. *Schizophr Bull*. 2003;29(4):703-715. doi:10.1093/oxfordjournals.schbul.a007040
